# A conversational multi-agent AI system for automated plant phenotyping

**DOI:** 10.1038/s41467-026-71090-y

**Published:** 2026-04-03

**Authors:** Feng Chen, Ilias Stogiannidis, Andrew Wood, Danilo Bueno, Dominic Williams, Fraser Macfarlane, Bruce D. Grieve, Darren Wells, Jonathan A. Atkinson, Malcolm J. Hawkesford, Stephen A. Rolfe, Tracy Lawson, Tony Pridmore, Sotirios A. Tsaftaris, Mario Valerio Giuffrida

**Affiliations:** 1 Institute for Imaging, Data and Communications (IDCOM), School of Engineering, University of Edinburgh, Edinburgh, UK; 2 James Hutton Institute, Dundee, UK; 3 Department of Electrical and Electronic Engineering, University of Manchester, Manchester, UK; 4 School of Biosciences, University of Nottingham, Nottingham, UK; 5 Rothamsted Research, Harpenden, UK; 6 School of Biosciences, University of Sheffield, Sheffield, UK; 7 Department of Plant Biology and Department of Crop Sciences, University of Illinois Urbana-Champaign, Urbana, IL USA; 8 Institute for Genomic Biology, University of Illinois Urbana-Champaign, Urbana, IL USA; 9 School of Life Sciences, University of Essex, Colchester, UK; 10 School of Computer Science, University of Nottingham, Nottingham, UK; 11 Causality in Healthcare AI Hub (CHAI), Edinburgh, UK

**Keywords:** Natural variation in plants, Optical imaging, Information technology, Machine learning

## Abstract

Plant phenotyping increasingly relies on (semi-)automated image-based analysis workflows to improve its accuracy and scalability. However, many existing solutions remain overly complex, difficult to reimplement and maintain, and pose high barriers for users without substantial computational expertise. To address these challenges, we introduce PhenoAssistant: a pioneering AI-driven system that streamlines plant phenotyping via intuitive natural language interaction. PhenoAssistant leverages a large language model to orchestrate a curated toolkit supporting tasks including automated phenotype extraction, data visualisation and automated model training. We validate PhenoAssistant through several representative case studies and a set of evaluation tasks. By lowering technical hurdles, PhenoAssistant underscores the promise of AI-driven methodologies to democratising AI adoption in plant biology.

## Abstract (web summary)

Application of large language models (LLMs) for automating complex and data-intensive crop phenotype analysis remains unexplored. Here, the authors integrate LLM with curated toolkit for phenotype extraction, visualization, and model training and show its ability to reduce technical barriers and enhance accessibility.

## Introduction

Plant phenotyping aims to quantify functional and structural traits (phenotypes) of crops and plants which result from complex interactions between genetics and environmental factors^1^. Phenotype analysis allows breeders and researchers to disentangle genetic effects from environmental adaptation enabling the development of crops with improved yields and climate resilience—an urgent need given that the global population is projected to reach 9.7 billion by 2050^2^ and extreme weather events become increasingly frequent^3^.

Accurate plant phenotyping often relies on computational workflows that chain multiple software tools for image processing to measure plant traits and subsequently perform data analysis. However, the steep learning curve associated with these workflows, such as programming, machine learning and data science, may prevent plant phenotyping researchers and practitioners from fully leveraging them^4^. The complexity of operating several software tools across different platforms present additional communication obstacles for both intra and inter-domain collaboration (e.g. between plant scientists with different specialisations, or between plant scientists and data scientists). Moreover, existing workflows typically employ fixed pipelines, which are difficult to extend or modify, restricting their applicability to broader tasks and scenarios. Despite calls for more accessible and versatile phenotyping systems^5^, this need remains inadequately addressed.

A promising approach to lowering technical barriers bridging domain expertise, and enabling more flexible phenotype analysis is by harnessing the power of large language models (LLMs)^6,7^. LLMs have emerged as powerful tools capable of solving diverse tasks from natural language instructions. Rather than focusing on developing computational skills, researchers can focus on critical scientific discovery and exploration by simply prompting an LLM to address the necessary computational and analytical tasks. However, this promise faces the challenge that LLMs alone often lack the specialised domain knowledge and requisite computational workflows to fulfil user-specified tasks^8,9^. An effective strategy is to augment LLMs with instructions and external tools forming AI agents capable of automatically executing tailored computational routines and data analysis pipelines.

An AI agent is a goal-directed software designed to perform tasks within a defined digital environment^10,11^. LLMs have recently become the core intelligence for many such agents (and hence also termed LLM agents) due to their strong abilities in natural language understanding and reasoning. These agents can receive task instructions, interpret and reason over inputs, and act to achieve specific objectives. They can also utilise external tools such as search engines or databases to extend their abilities^12^. A multi-agent AI system extends this paradigm by integrating several specialised LLM agents and tools within a coordinated framework, enabling division of labour across subtasks and allowing the system to address broader and more complex tasks than a single agent could manage alone^11^. Agentic AI represents multi-agent AI systems that exhibit higher adaptability and autonomy. Rather than operating with fixed roles, such systems often allow agents to interact with each another flexibly, negotiate responsibilities, and adapt their behaviour to dynamic contexts^12–14^. These different levels of complexity and flexibility distinguish the three paradigms and make them suitable for different scenarios: AI agents can operate independently on single bounded tasks; multi-agent AI systems are well suited for tasks requiring multi-step workflows; and agentic AI leverages emergent behaviours to support more exploratory or creative tasks.

Recently, LLM-based agent systems have successfully been applied across different scientific domains, including chemistry^15,16^, material sciences^17,18^, biology^19,20^ and healthcare^21–23^. They not only reduce the technical difficulties of domain-specific computation and data analysis, but also serve as effective platforms for multidisciplinary collaboration. Experts from diverse fields can contribute to the analytical process by interacting with the system through natural language. For example, a plant scientist unsure about which data analysis approach to use could consult a data scientist, then relay the detailed instructions to the AI system to automate the task. Feature comparison of several existing LLMs and LLM-based agent systems for plant science and agriculture is presented in Supplementary Table 1. While LLMs and LLM-based agent systems have also been applied to agriculture and plant sciences (e.g. agricultural Q&A^24–28^, disease and stress phenotyping^29–32^, genome prediction^33–35^), their potential for automating complex and data-intensive phenotype analysis workflows remains underexplored.

In this work, we introduce PhenoAssistant, an open-source multi-agent AI system for automating plant phenotype analysis. By integrating a generalist LLM with a specialised toolkit for plant research, PhenoAssistant allows plant scientists to use free-text task descriptions to prompt a wide range of phenotyping-related data analysis pipelines, such as phenotype extraction from images, phenotypic statistics analysis, and data visualisation. The toolkit features cutting-edge deep learning models and LLM agents to support users’ dynamic needs in extracting and analysing plant traits. PhenoAssistant can be extended (e.g. through built-in model training functions) to accommodate customised and emerging needs. To improve reproducibility, it also allows users to save the generated pipelines and re-execute them on similar datasets. Overall, PhenoAssistant serves as a pioneer for leveraging multi-agent systems in plant phenotyping. We demonstrate its potential to streamline complex workflows and democratise AI adoption in plant biology through three case studies and evaluations covering three aspects. By lowering technical barriers and facilitating collaborations across different areas of expertise, PhenoAssistant highlights the promising future of LLM-based agent systems in accelerating scientific discovery in plant science.

## Results

### Design of PhenoAssistant

**Fig. 1:**
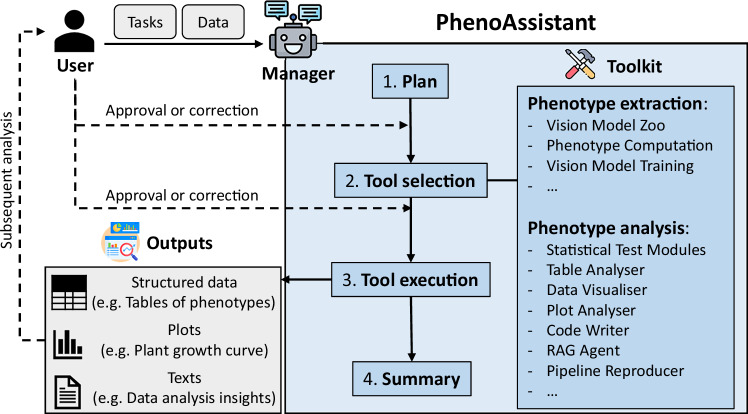
Design of PhenoAssistant. Users provide data and task description to PhenoAssistant. The manager creates a step-by-step plan selects and executes appropriate tools, and then summarises the tool outputs to fulfil the task. Users retain full control to refine intermediate steps as needed. Manager icon made by Surang from Flaticon (www.flaticon.com). Outputs and Toolkit icons made by Freepik from Flaticon (www.flaticon.com).

The goal of PhenoAssistant is to generate and execute suitable workflows by chaining AI models and other computational tools to address plant phenotype analysis requests from users. To achieve this, PhenoAssistant is built around a centralised multi-agent architecture, comprising an augmented LLM (the manager) and a specialised toolkit consisting of dedicated computational modules and LLM agents designed to enhance its capabilities for image-based plant phenotyping, as illustrated in Fig. 1.

In this architecture, a single manager agent is responsible for coordinating workflow creation and execution. The role of each LLM agent in the system is pre-defined and fixed. This architecture is chosen because it best supports our aim of reliable workflow generation and execution. It offers several advantages for plant phenotype analysis: it is less prone to coordination errors, produces more consistent workflows for similar task descriptions, and operates within a clearly defined set of available tools and agents. This architecture also increases transparency and user control by allowing users to intervene at any step of the generated workflow to correct errors or unexpected outcomes simply by interacting with the manager.

Specifically, upon receiving user-provided task descriptions and accompanying data, the manager first creates a plan detailing the necessary steps to fulfil the task. It then selects and executes the suitable tools with appropriate parameters, producing various types of outputs that can be saved for further analysis (either by the user or PhenoAssistant). Finally, the manager summarises these outputs, presenting essential information for task completion. Users retain control throughout this process, and can provide textual feedback to refine plans or select alternative tools and parameters, minimising errors from PhenoAssistant.

A core component of many plant phenotype analysis workflows is accurate visual information extraction. While recent generalist LLMs and agents often include vision capabilities^6,36,37^, they are not always able to generalise to extracting plant phenotypes due to the limited representation of plant-specific data and tasks during their development. To demonstrate this limitation, we prompt the state-of-the-art generalist agent, ChatGPT 4o, in various ways, to extract the projected leaf area (PLA) and leaf count from an image sourced from the same dataset that will later be used in case study 1. The actual PLA and leaf count for this image are 14,260 pixels and 14, respectively.

**Fig. 2:**
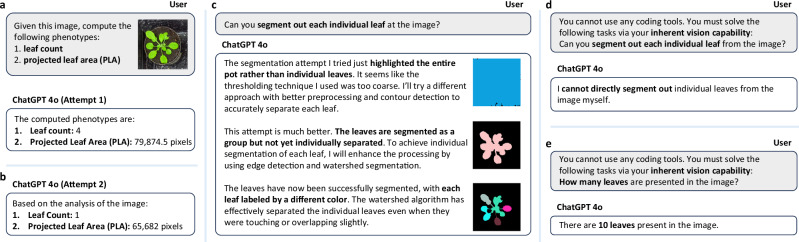
Examples of using ChatGPT 4o to extract plant phenotypes and perform computer vision tasks on a top-view image of *A. thaliana.* **a**, **b** ChatGPT is prompted to extract projected leaf area and leaf count in two separate attempts, but the results are incorrect and inconsistent between attempts. **c** ChatGPT is prompted to perform leaf instance segmentation. It gradually refines its segmentation approach from producing no meaningful output, to generating a rough plant mask, and finally several leaf masks. However, the final output still fails to separate each leaf individually with clear boundaries. ChatGPT is prompted to rely solely on its inherent vision capabilities to perform leaf instance segmentation (**d**) and leaf counting (**e**). However, these capabilities do not support instance segmentation and also produce an incorrect leaf count. The same *A. thaliana* image from (**a**) is used as visual input for all examples. Screen captures of user-ChatGPT conversations corresponding to these examples are provided in Supplementary Fig. 1.

As illustrated in Fig. 2a, ChatGPT is directly prompted to extract the requested phenotypes without additional instructions. However, it cannot correctly predict either the leaf count or PLA, despite it invokes the code writing function, attempting to use image processing techniques to solve the tasks. Furthermore, ChatGPT produces inconsistent results when processing the same prompt repeatedly (Fig. 2b). Next, as shown in Fig. 2c, we prompt ChatGPT to perform a computer vision pre-task (leaf instance segmentation), which allows for manual extraction of PLA and leaf count from the segmentation results. However, the results are inadequate for further analysis: several edges are incorrectly segmented, and not all individual leaves are successfully identified. Finally, we prompt ChatGPT to rely solely on its vision capabilities to extract the phenotypes, but the results are still unsatisfactory: tasks, such as leaf instance segmentation, cannot be performed using ChatGPT’s vision abilities (Fig. 2d), and leaf count predictions are incorrect (Fig. 2e).

To overcome this limitation in generalist LLMs and agents, PhenoAssistant is equipped with phenotype extraction tools, incorporating a model zoo of computer vision models trained on plant-specific datasets, with utilities for computing traits, such as plant area and diameter, from these models’ outputs. The toolkit also supports automatic model training on new data, enabling users to expand PhenoAssistant’s vision capabilities to their unique data and analytical requirements.

Complementing these phenotype extraction tools, PhenoAssistant is also augmented with tools for phenotype analysis. This includes dedicated modules with pre-defined logic and deterministic processes for statistical tests (e.g. ANOVA). In addition, the toolkit contains various LLM agents engineered for different purposes, including the table analyser for extracting information from CSV files, the data visualiser for generating plots, and the plot analyser for interpreting and analysing generated plots. Additionally, the code writer agent supports essential tasks necessary for developing comprehensive end-to-end analysis workflows, such as merging and saving files, as well as executing novel functions not predefined within the toolkit. To enhance PhenoAssistant’s expertise in plant-related domains, a retrieval augmented generation (RAG) agent provides access to scientific literature. The Pipeline Reproducer agent enables users to extract and re-execute previously conducted phenotype extraction and analyses, including all tool calls and generated code, ensuring reproducibility for similar data.

### Case studies

Traditionally, executing plant phenotyping tasks requires substantial expertise in computer science, including image processing, machine learning, and coding. With PhenoAssistant, users can complete these tasks effortlessly by interacting through natural language. To demonstrate PhenoAssistant’s effectiveness, we present three case studies showcasing its ability to streamline plant phenotyping analysis.

In case study 1, we use PhenoAssistant to replicate part of the Phenotiki analysis workflow^38^, aiming to visualise and analyse the growth patterns of different *Arabidopsis thaliana* ecotypes. We provide PhenoAssistant with the same dataset as in Minervini et al.^38^, which contains 24 plants from 5 different ecotypes: wild type (Col-0, 5 plants), constitutive triple response 1 (*ctr1*, 5 plants), ethylene insensitive 2 (*ein2.1*, 5 plants), *pgm* (mutation in the plastidic isoform of phosphoglucomutase, 5 plants), and *adh1* (mutation causing defects in alcohol dehydrogenase, 4 plants). Each plant was grown for 26 days with images captured every 12 h, resulting in a total of 1248 images.

**Fig. 3:**
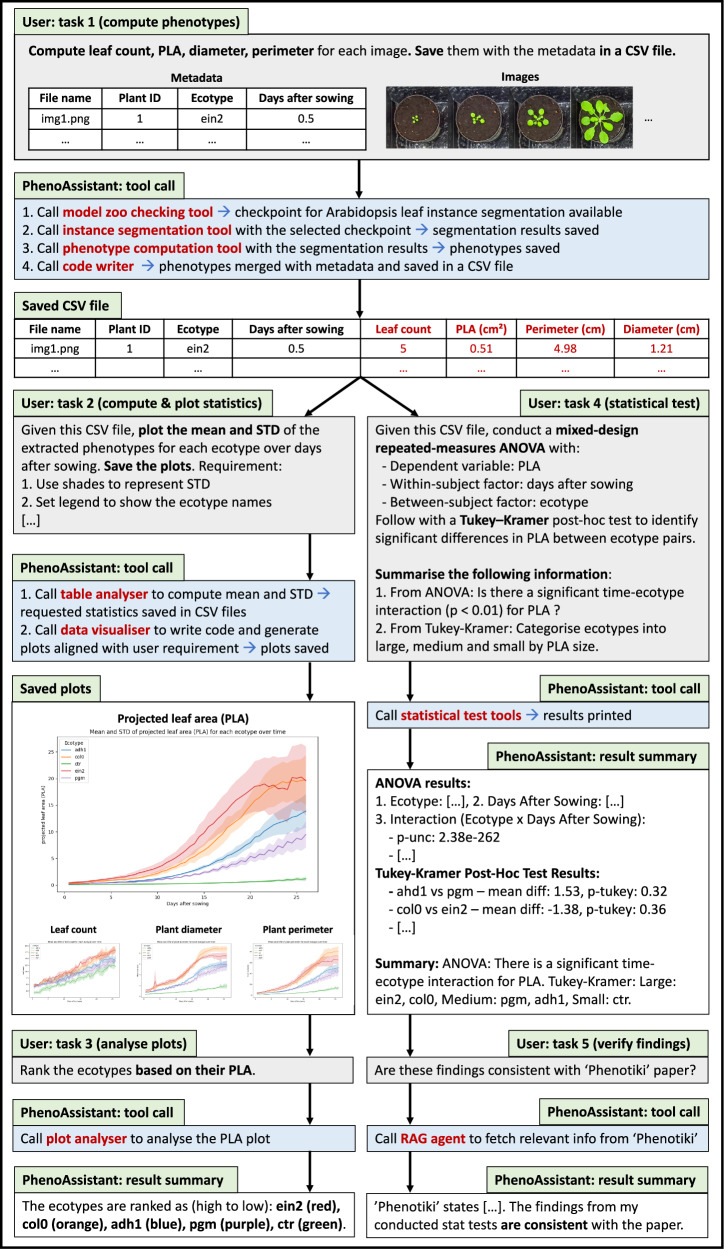
Case study 1—*A. thaliana* growth pattern analysis. PhenoAssistant automatically completes five tasks: computing phenotypes from images, plotting phenotypic statistics, analysing a generated plot, performing statistical tests for different ecotypes, and comparing findings with literature. Each task is presented as task description (grey), tools used by PhenoAssistant (blue), and results (white). Enlarged versions of the saved plots in task 2 are provided in Supplementary Figs. 2–5.

As shown in Fig. 3, we prompt PhenoAssistant to address five relevant tasks, each including a task description, tools used by PhenoAssistant, and the generated results. We begin with the fundamental step of any plant phenotyping task, extracting phenotypes from data: in task 1, PhenoAssistant is prompted to extract phenotypes including projected leaf area (PLA) and leaf count from images. It identifies the need for a leaf instance segmentation model and hence selects the one suitable for *Arabidopsis* from the model zoo, executes it, and computes the requested phenotypes from the segmentation results. The code writer is then employed to write and execute Python code to merge the computed phenotypes with metadata and save them into a CSV file. To illustrate how users can interact with PhenoAssistant in practice, we provide the chat logs for case study 1 task 1 in Supplementary Note 1. At the end of task 1, the user can request PhenoAssistant to save the executed pipeline (i.e. from instance segmentation to saving the extracted phenotypes, as demonstrated at Supplementary Note 2), which can later be reapplied to a different dataset.

From tasks 2 to 5, we demonstrate PhenoAssistant’s capabilities for processing and analysing the extracted phenotypes (i.e. from task 1), which are essential for identifying plant growth trends and key differences among ecotypes. In task 2, PhenoAssistant leverages the table analyser and data visualiser agents to compute and plot statistics based on user-specified plot requirements to visualise the growth patterns of different ecotypes. It can further use the plot analyser to interpret data visualisations, such as ranking ecotypes according to their PLA (Task 3). PhenoAssistant can also invoke relevant tools to conduct statistical analyses (e.g. ANOVA and Tukey–Kramer) using user-defined parameters and then summarise the key findings (task 4). For example, it categorises ecotypes by PLA size as large (*ein2.1*, Col-0), medium (*adh1*, *pgm*), and small (*ctr1*), based on Tukey–Kramer post hoc tests (*p* < 0.05). Furthermore, PhenoAssistant can validate these findings against previous literature by leveraging the RAG agent to embed paper contents and retrieve related knowledge (task 5). In tasks 4 and 5, PhenoAssistant performs the same statistical tests and reaches conclusions consistent with the Phenotiki paper^38^.

In case study 2, we show that PhenoAssistant can handle other data (e.g. non-model plant) and tasks when provided with appropriate tools, such as vision models developed on other datasets. To illustrate this flexibility, we prompt PhenoAssistant to replicate part of the workflow in Williams et al.^39^ for potato leaves, assessing the correlation between manually measured leaf area ($A$) and dry weight ($W$), and evaluating if this correlation holds with algorithmically computed PLA. This analysis can help plant scientists identify potential discrepancies between manual and automated measurements. For this demonstration, we integrate the leaf-only SAM model (for potato leaf segmentation) and use a dataset consisting of 32 potato images with $A$ and $W$ annotated; both the model and dataset originate from Williams et al.^39^.

**Fig. 4:**
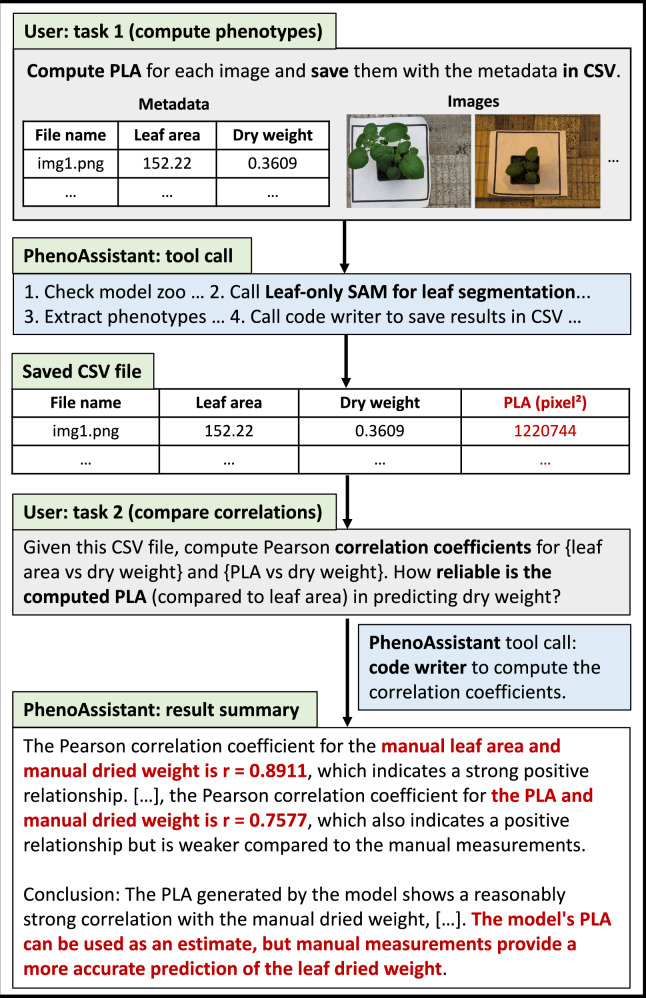
Case study 2—potato leaf area and dry weight correlation analysis. In response to the user’s requests, PhenoAssistant first extracts phenotypes from the provided data and then compares correlations between different plant-related variables.

As shown in Fig. 4, PhenoAssistant is asked to compute PLA, and it follows the similar procedure to the previous case study while using a different segmentation model (task 1). It is then tasked to measure and analyse the Pearson correlation coefficient between $A$ and $W$, as well as PLA and $W$ (task 2). As this correlation analysis is not predefined in the toolkit, PhenoAssistant leverages the code writer to accomplish it. Finally, PhenoAssistant summarises the results, highlighting the correlation coefficients and providing further explanations and context. The findings indicate that while the PLA derived from leaf-only SAM is useful, it may introduce errors compared to manually measured leaf area when predicting dry weight. In task 2, PhenoAssistant performs the same correlation analysis and reaches conclusions consistent with Williams et al.^39^.

In case study 3, we demonstrate that PhenoAssistant can expand its vision capabilities via the built-in automatic vision model training function, enabling it to tackle previously unseen phenotyping challenges (e.g. experiments with new data or in new environments).

**Fig. 5:**
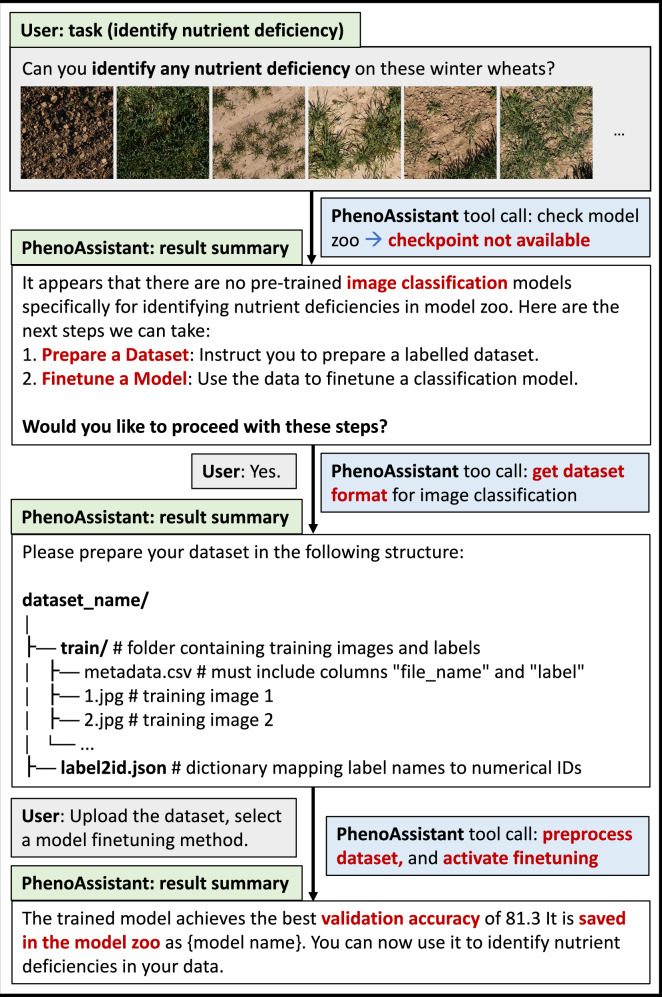
Case study 3—automatic model training for nutrient deficiency identification. When no suitable model is available to solve a given task, PhenoAssistant first prompts the user to provide a dataset in the desired format. The user can select between full-parameter finetuning or LoRA^40^, depending on computational resource constraints and performance requirements. It then automatically applies data preprocessing, followed by training and evaluating the model. The trained model is saved in the model zoo for future use.

As shown in Fig. 5, PhenoAssistant is prompted to identify nutrient deficiency for winter wheats in field, where no capable model is integrated in the toolkit. Recognising the need for image classification, it instructs the user to upload labelled data in the required format. After receiving the dataset, it invokes tools to automatically preprocess the data (e.g. splitting into training and evaluation subsets, applying data augmentation), initiate model training using the user-specified finetuning method (full-parameter finetuning or low-rank adaptation (LoRA)^40^, whose differences are described in the automatic vision model training subsection of the 'Methods' section), and evaluate the model’s performance. Finally, the evaluation results are presented to the user, and the trained model is added to the model zoo for future use. The data used for this case study are from Yi et al.^41,42^.

In summary, we present three case studies illustrating how PhenoAssistant streamlines plant phenotyping with natural language instructions, allowing plant researchers to concentrate on scientific discovery rather than technical training. These cases span various plant species, environmental conditions, and tasks, including phenotype extraction, pipeline reproduction, statistics computation and visualisation, plot analysis, statistical testing, knowledge retrieval, correlation assessment, and model training. Chat logs for all case studies are available in our code repository.

### Evaluation of PhenoAssistant

Beyond the presented case studies, we evaluate PhenoAssistant’s adaptability and potential for broader applications via a series of tasks. Since PhenoAssistant leverages rapidly evolving AI technologies, this evaluation also offers a method for assessing new AI components (e.g. DeepSeek^43,44^) as replacements for PhenoAssistant’s original modules. The evaluation covers three aspects: tool selection, vision model selection, and data analysis. Prompts and detailed results for the evaluation are presented at Supplementary Notes 3–5. Chat logs for the evaluation are available in our code repository. During the evaluation, no human feedback (e.g. corrections to tool selection or tool arguments) is provided.

We evaluate the tool selection ability of PhenoAssistant by assessing whether it can select appropriate tools with the correct parameters and in the correct order to complete a task. This evaluates PhenoAssistant’s abilities of chaining multiple tools for task completion rather than the correctness of task outputs.

To conduct this evaluation, we create 20 tasks to represent various phenotyping requests with different plant species. For each task, we prompt PhenoAssistant to generate a sequence of tools with their arguments in a standalised format. The outputs from PhenoAssistant are evaluated by an LLM-based evaluator, termed tool evaluator (LLM-based evaluators have been applied in different domains^15,45^). The tool evaluator is an LLM (GPT-4o) provided with detailed information about PhenoAssistant’s toolkit (e.g. descriptions of tool functionalities and arguments) and the user-specified task description. It is prompted to score the response of PhenoAssistant for each task on the following four aspects using a scale of 1–5 (where 1 is the lowest and 5 the highest):

1. Overall chain: whether the tool chain is reasonable for addressing the task and whether any crucial steps are missing or incorrectly ordered.
2. Tool existence: whether each selected tool exists within PhenoAssistant’s toolkit.
3. Tool appropriateness: whether each tool is suitable for the specific step where it is applied.
4. Arguments: whether the arguments for each tool are correctly specified.

The average scores across the 20 tasks are reported in Table 1, under the row manager. Overall, PhenoAssistant consistently achieves a full score in tool existence correctness (5), indicating that it does not hallucinate tools outside its toolkit. Slightly lower scores in overall chain correctness (4.25) and argument correctness (4.3) suggest that PhenoAssistant might generate toolchains in an inappropriate order, omit certain steps, or provide less accurate arguments. These issues may also be caused by vague task descriptions, highlighting the importance of allowing users to clarify tasks through interaction with PhenoAssistant. PhenoAssistant also performs well on tool appropriateness (4.65), although this score still indicates potential limitations in the tool understanding abilities of LLMs, as well as in the clarity and comprehensiveness of the tool descriptions provided within the toolkit in PhenoAssistant.

**Table 1:** Tool selection evaluation results under two settings (manager and manager + critic)

Setting	Overall chain	Tool existence	Tool appropriateness	Argument	Average
Manager	4.25	5.00	4.65	4.30	4.55
Manger + critic	4.35	5.00	4.90	4.40	4.66

For each setting, the scores at each column are averaged across 20 tasks.

Beyond evaluating the original design of PhenoAssistant, we also assess the potential of introducing a critic agent. This agent is implemented as a prompted LLM (GPT-4o), provided with both the user-specified task and PhenoAssistant’s toolkit information, to critique and refine the toolchains generated by the manager. Each toolchain produced by the manager is reviewed by the critic and will be refined if issues are identified. The toolchains generated by the manager–critic interactions are assessed with the same tool evaluator in the manager-only setting.

The average scores with the critic are presented in Table 1 under the row manager + critic. The results show consistent improvements across all aspects compared to using the manager solely. This suggests that strategy verification mechanisms (e.g. adding a critic agent) can enhance the reliability of PhenoAssistant on broader tasks and reduce the burden and frequency of human verification, which should be considered in future extensions.

The system prompts for the tool evaluator and the critic are provided in Supplementary Note 6. Detailed per-task scores for the tool selection evaluation are reported in Supplementary Table 2.

Next, we evaluate whether PhenoAssistant can recommend the appropriate type of computer vision model (instance segmentation, image classification, or image regression) for a given plant phenotyping task. We term this evaluation vision model selection I. It is crucial for automatic model training, as users may not have substantial machine learning knowledge to associate plant tasks with computer vision models. Accurate suggestions from PhenoAssistant prevent users from preparing incorrect training data.

To conduct this evaluation, we create 50 plant tasks expressed in varied formats: short phrases (e.g. leaf counting), statements (e.g. highlight the weeds in these field images), and questions (e.g. what is the maturity status of these pods?). This variation aims to cover different ways users might naturally express tasks. PhenoAssistant is then asked to select the most appropriate model type corresponding to each task description. The outputs of PhenoAssistant are manually inspected.

PhenoAssistant achieves 100% accuracy in vision model selection I, indicating its potential to activate the correct model training function for a wide range of plant tasks.

In addition to evaluating PhenoAssistant’s ability to select the correct model type, we further assess whether it can identify the most suitable specific model from a vision model zoo for a given plant phenotyping task. We term this evaluation vision model selection II. It is designed to test whether PhenoAssistant can handle broader phenotyping scenarios when its model zoo expands.

To perform this evaluation, we adapt tasks from vision model selection I and create 20 tasks covering a wide range of plant species and phenotyping needs (e.g. identify all the wheat spikes in these images, and how badly are these sunflowers affected by disease? Provide a score for each image). We also create a vision model zoo containing 30 model identifiers. Each identifier follows the same naming convention used in the vision model zoo of PhenoAssistant, namely {plant-species}_{task}_{model} (e.g. wheat_spike-instance-segmentation_m2fb, sunflower_disease-severity-regress_dino2b), as described in the vision model zoo subsection of the 'Methods' section. Among these 30 model identifiers, 15 identifiers correspond exactly to 15 of the 20 plant tasks, while the remaining 15 identifiers serve as distractors.

PhenoAssistant is then asked to match each plant task with the most suitable model identifier from the model zoo. It achieves 100% accuracy in this evaluation: for the 15 tasks with corresponding model identifiers, it provides the correct match; for the remaining 5 tasks without a direct match, it not only recognises that no suitable model is available, but also recommends an appropriate model type for fine-tuning. This result demonstrates PhenoAssistant’s potential to address a wider range of plant phenotyping tasks with an extended vision model zoo.

Finally, we assess whether PhenoAssistant can produce the correct outputs for different data analysis requests. This aims to assess the reliability of the built-in LLM agents (e.g. data visualiser) for data analysis.

To conduct this evaluation, we create 20 phenotype analysis tasks using actual data. These tasks cover a range of operations including extracting values from files, computing statistics, generating plots, analysing plots, converting file formats, computing and analysing new metrics. The outputs of PhenoAssistant are manually inspected.

PhenoAssistant achieves 85% (17/20) accuracy in this evaluation. All failed cases fall within the plot analysis category. These tasks require the use of the plot analyser agent (a prompted GPT-4o) to interpret and extract fine-grained information from figures. This suggests that fine-grained visual reasoning remains a bottleneck for LLMs.

In summary, our evaluation shows that PhenoAssistant, as a multi-agent AI system, exhibits promising adaptability to broader plant phenotyping scenarios and tasks, while strategy verification mechanisms and improved visual interpretation capabilities should be further explored.

## Discussion

In summary, we introduce PhenoAssistant, a pioneering multi-agent AI system designed to streamline data-intensive and complex workflows for plant phenotyping researchers and practitioners while reducing the requirements for computational expertise and the burden of programming. By integrating cutting-edge AI technologies, PhenoAssistant transforms simple, interactive conversations into complex image-based plant analyses. Our case studies demonstrate its versatility across a range of phenotyping tasks and plant species, while evaluations highlight its potential of extending to broader scenarios. Overall, PhenoAssistant marks an important step toward democratising AI adoption in plant phenotyping and lays a solid foundation for future AI-driven advancements in plant science. To facilitate ongoing development and validation, PhenoAssistant is made available as open source.

Despite its success, PhenoAssistant has limitations. Due to current LLMs’ constraints in understanding and decomposing complex tasks, some tasks may need to be carefully instructed or broken down into multiple steps. This can be improved with more effective prompt engineering or fine-tuning on LLMs. We also integrate a function to reproduce executed pipelines, reducing the user-LLM interaction for these complex tasks.

In addition, our evaluations reveal problems on tool selection and data analysis, indicating that a certain level of human inspection of the generated workflows and results is still required. We show that introducing an additional critic agent to verify the responses generated by the manager agent can improve tool selection. In the future, other strategy verification mechanisms, such as sandboxing and agent self-evaluation strategies should also be explored^46,47^. Although the current evaluations cover the essential functions of PhenoAssistant, they still require a degree of human involvement to design the evaluation tasks and perform the assessments. This may limit the scalability of evaluations when extending PhenoAssistant to a larger set of tools or tasks. Future work should aim to automate evaluations to enhance scalability.

PhenoAssistant currently relies on users to provide information about their data source or inspect the extracted phenotypes to mitigate the risks of selecting and applying vision models to conditions different from those they were originally trained on (e.g. variations in imaging setups, lighting, or growth environments). This limitation can be addressed by using the integrated training function to develop new models on users’ own datasets; looking ahead, it could be further reduced by integrating out-of-distribution (OOD) detection mechanisms^48,49^ or vision foundation models capable of handling more general agricultural and plant conditions^50,51^.

PhenoAssistant’s adaptability to new tasks and scenarios is mainly constrained by the coverage and capabilities of its toolkit. To extend vision capabilities, PhenoAssistant supports automatic model training, but this requires users to collect and label the training data. Meanwhile, AI community platforms (e.g. Hugging Face^52^ and Kaggle^53^) host many trained computer vision models for plant-related tasks. In the future, dynamically fetching suitable models from these platforms based on user-specified plant species and phenotyping tasks should be explored to further reduce user effort.

However, several requirements must be met before such a mechanism can be reliably integrated. First, model identifiers and descriptions on these platforms should be standardised and made more comprehensive, so that the training data sources as well as plant species and phenotypic traits to which each model can be applied, are clearly specified and easily retrieved. Second, mechanisms are required to assess compatibility between fetched models and user-provided data, since models may fail when applied to scenarios different from those they were originally trained on. Third, external model repositories should ensure long-term stability through consistent access protocols and transparent versioning, mitigate issues arising from model removal or changes in authentication and data formats. Finally, safeguards are needed to ensure that dynamically fetched models are free from malicious components or adversarial behaviours that affect the safety and robustness in plant phenotyping.

In addition, standards such as model context protocol (MCP)^54^ should be explored to allow users integrate other tools more easily. These standards enable software developers to make their products readily integrable with LLMs, fostering a growing ecosystem of LLM compatible tools with diverse functionalities. As the needs and interests of applying LLM agent systems in plant phenotyping grow, more related tools will adopt such standards and make this form of extension more practical.

While PhenoAssistant focuses on streamlining phenotype extraction and analysis, plant phenotyping (more broadly, plant science and agriculture) encompasses other tasks that could benefit from LLM agents. For example, simulating plant-related hypotheses (e.g. responses to different nutrient deficiencies under different conditions) could help researchers narrow down experimental options, reduce risks, and ultimately save resources and time before deploying costly real-world experiments. One emerging direction is agent-based simulation, where agents explore what-if scenarios to probe underlying mechanisms and assess potential outcomes by mimicking real-world processes. Such approaches have been investigated in domains such as economic systems^55,56^ and physical environments^57,58^, though challenges in efficiency and robustness remains insufficiently addressed^59^.

Closely aligned with the goals of simulation, reasoning about cause–effect relationships in plant physiology can help researchers formulate mechanistic hypotheses and predict phenotypic responses to novel environments or management strategies. Although PhenoAssistant already demonstrates abilities in statistical testing, literature-based verification and correlation analysis (case study 1 and 2), it does not explicitly support causal modelling. Recently, agent-based causal reasoning and validation has emerged as an active research area^60,61^. However, existing studies also identify that current agent-based causal modelling often lacks deep causal understanding and are limited to shallow, low-level reasoning^12,62^.

PhenoAssistant adopts a centralised multi-agent architecture where a single manager coordinates tools and agents with pre-defined roles to accomplish user-specified tasks. This architecture is well-suited to the current objective on plant phenotyping workflow generation and execution. However, it may limit an agent system’s capacity for emergent intelligence and constrain its ability to address more open-ended or creative tasks. Recent research on agentic AI (i.e. more autonomous and self-adapting multi-agent AI system) investigates decentralised designs in which different agents interact more flexibly through peer-to-peer communication with shared task contexts^63,64^; this trend is further supported by emerging multi-agent frameworks^65,66^. Alongside this, dynamic role negotiation has emerged as an alternative to static role assignments, allowing agents to self-assign responsibilities and adapt them in response to evolving task requirements^67,68^. Such advances in agent orchestration and communication are promising for fostering emergent behaviours and scalability^12^, and hence are suitable for tackling exploratory tasks in agriculture and plant science beyond workflow automation, such as proposing novel research directions or unconventional experimental designs. At the same time, decentralised coordination and dynamic role assignment introduce challenges, such as longer inference time due to negotiation overhead, instability or inefficiency when roles are poorly defined, and additional safety and governance concerns^12,69^.

To address these concerns and extend multi-agent systems such as PhenoAssistant more broadly, recent surveys^12,69^ suggest that proper scalable infrastructure and governance are required. Scalable infrastructure should include flexible agent orchestration mechanisms (from centralised managers with fixed-role agents to decentralised agents with dynamic role negotiation). It should also provide standard protocols for integrating external tools (e.g. MCP as mentioned above) and allowing agents built from different frameworks to interact securely and effectively (e.g. Agent2Agent protocol^70^). In addition, it should include robust resource scheduling and conflict handling mechanisms between agents to prevent potential bottlenecks and instability of agent systems. Moreover, a shared memory layer (e.g. vector databases) is important to providing different agents with a consistent and queryable context on task progress, while observability pipelines that log conversation histories and tool use are crucial for human users to audit and debug.

Lastly, complementing scalable infrastructure, governance policies are required to keep multi-agent AI systems safe and accountable as they scale. Mechanisms for automatic or manual supervision are needed to ensure each agent has transparent responsibility, aligns to the overall goal, and accountable for its subtasks. Access controls and sandboxing protect privacy and safety when agents interact with external data and tools. Configurable memory retention policies (e.g. for vector databases) can prevent unintended leakage of task execution context. Finally, certain level of human-in-the-loop involvement should be preserved to trace agent actions and intervene if problems arise, such as biased interpretation of data or agents drifting away from the intended objectives.

## Methods

### LLMs and agents

LLMs are deep neural networks with billions of parameters trained on large-scale text corpora to predict the next token in a sequence. Although this objective is simple, it enables LLMs to acquire emergent abilities, such as natural language understanding, reasoning and code generation. Recent models such as GPT-4o^6^, have also extended to multi-modal training (e.g. using both text and image data), and hence expanded their capabilities to vision-language interpretation, making them suitable to support more complex tasks.

The versatile abilities of LLMs make them effective foundations for AI agents (and hence also termed LLM agents). These agents leverage LLMs as core intelligence for reasoning, decision-making, and performing actions to achieve specified goals, such as operating structural data or retrieving knowledge from literature. This is typically achieved by providing an LLM with (i) a carefully crafted system prompt, which defines the agent’s role and acting logics (e.g. to follow specific instructions or avoid common mistakes), and (ii) access to external tools, such as computational functions, APIs, or databases, which extend the abilities of an LLM beyond those acquired from model training. When multiple agents and tools with specialised functionalities are integrated into a multi-agent AI system, the system as a whole can collectively address complex tasks that exceed the capacity of a single agent. To ensure that a such system aligns with its objectives, both system-level design (e.g. incorporating human-in-the-loop mechanisms) and element-level design (i.e. selecting appropriate agents and tools) need to be carefully considered.

### Implementation of PhenoAssistant

PhenoAssistant comprises a manager agent and a specialised toolkit designed for plant phenotyping tasks. The manager provides an interactive interface for handling user requests, planning tasks, selecting and executing tools, and summarising results. The manager is implemented using GPT-4o (version: 2024-08-06, via OpenAI Azure API), which was the state-of-the-art LLM when PhenoAssistant was developed, with the model temperature set to 0.1, guided by a system prompt (Supplementary Note 6) that instructs it to ensure desired functionality and behaviour.

Specifically, the system prompt instructs the manager agent to (i) generate a step-by-step plan and refine it iteratively, (ii) coordinate the use of the toolkit by chaining outputs and inputs across different tools and agents, (iii) follow specialised guidelines when using specific tools or agents (e.g. the manager must verify the availability of a vision model before invoking it), and (iv) conclude interactions with a concise summary.

The toolkit consists of Python-based functions, each accompanied by structured schema (name, description, parameters, input/output format). This enables the manager agent to understand the tools’ capabilities and compose them properly into valid workflows. Notably, PhenoAssistant’s toolkit includes both deterministic modules (e.g. instance segmentation and statistical tests) and LLM agents (e.g. code writer and plot analyser).

PhenoAssistant is implemented in Python, with tool invocation and multi-agent orchestration supported by AutoGen. Computer vision models and model training functions are implemented using PyTorch and the Transformers library. The table analyser agent is implemented using Pandas AI. We introduce the key tools below; other tools, as well as a complete list of software dependencies is available in our code repository.

### Vision model zoo

Many plant phenotypes cannot be extracted without the aid of image processing or computer vision techniques. Although recent LLMs have incorporated vision capabilities, they are not yet powerful enough for effective extraction of plant traits. For example, tasks such as delineating individual leaf boundaries (known as instance segmentation in computer vision), which is a preliminary task for computing leaf area and count, remain challenging.

To overcome this limitation, PhenoAssistant integrates external computer vision models. Each vision model is uniquely identified using the naming convention: {plant-species}_{task}_{(optional) training-dataset}_{model}_{(optional) finetuning-method}. These identifiers are maintained within a structured file (e.g. model_zoo.json), which the manager agent accesses whenever a vision model is required, ensuring appropriate vision model selection for the given plant task.

For demonstration purposes, in case study 1, we integrate Mask2Former^71^ for segmenting individual *Arabidopsis* leaves. Following the similar practice in Chen et al.^72^, Mask2Former is fine-tuned on subsets A1 and A4 of the publicly available CVPPP LSC dataset^73^. In case study 2, we integrate Leaf-only SAM^39^ for potato leaf instance segmentation.

### Automatic vision model training

Given the diverse conditions encountered in plant phenotyping, such as varying illumination, indoor/outdoor environments, species diversity and diverse phenotyping needs, it is practically impossible to pre-integrate every required vision model into PhenoAssistant. Currently, there is also no universal vision model capable of solving all phenotyping tasks. Furthermore, new tasks and customised user requirements continuously emerge, highlighting the need for a convenient mechanism to expand PhenoAssistant’s vision model zoo.

To satisfy this need, PhenoAssistant incorporates an automated model training pipeline for common phenotyping tasks (e.g. image classification). When users identify the need for a new vision model or when PhenoAssistant determines that its current model zoo cannot adequately solve a provided task, PhenoAssistant prompts the user to upload a dataset formatted according to predefined specifications. PhenoAssistant calls tools to automatically split the uploaded dataset into training and validation sets, as well as initiate model training. Upon completion, the newly trained model is automatically added to the vision model zoo using the naming convention described earlier and becomes available for inference.

The core idea behind this automated training pipeline is to fine-tune pre-trained vision models using plant-specific datasets^74^. These models are initially trained on large-scale general data and can be fine-tuned to specific plant analysis tasks with limited additional data, thereby minimising development and deployment efforts. Specifically, for image classification (as shown in case study 3), we employ the DINOv2-base model^75^ as the pre-trained model.

PhenoAssistant supports two fine-tuning strategies: low-rank adaptation (LoRA)^40^ and full fine-tuning, to accommodate users with varying levels of computational resources. LoRA updates only a small subset of parameters by inserting low-rank trainable matrices into existing model layers, substantially reducing computational and memory requirements during training. This approach enables rapid adaptation at the cost of a modest performance trade-off. In contrast, full fine-tuning updates all model parameters, potentially achieving higher accuracy while at increased computational and memory demands.

### LLM agents for phenotype analysis

Beyond phenotype extraction, PhenoAssistant supports diverse data analysis tasks, such as statistics computations, data visualisation, and interpretative plot analysis. These analyses often involve complex and customised requirements that cannot always follow pre-defined logic. For example, users may request specific visualisation styles (e.g. plotting plant growth curves for different ecotypes using specific colour) or novel analyses prompted spontaneously during exploration.

To support these dynamic and evolving analytical needs, we repurpose GPT-4o (same as the manager agent, version 2024-08-06 via the OpenAI Azure API with temperature set to 0.1) into different roles using different system prompts (Supplementary Note 6). This configuration creates a set of distinct LLM agents, each of which can be invoked by the manager when required:

1. Code writer: generates and executes Python code for performing tasks that are not previously defined in the toolkit. Code execution is performed with automatic error handling and retry logic. Its system prompt explicitly instructs the agent to output complete, runnable scripts.
2. Data visualiser: generates and executes Python code for producing plots with user specification. Guidance for creating clear and well-designed plots (e.g. including legends and beautifying layout) is provided within its system prompt.
3. Plot analyser: interprets plots using GPT-4o’s visual understanding capabilities.
4. Table analyser: queries values and computes statistics from CSV files using Pandas AI. Pandas AI is an agent framework that augments an LLM (GPT-4o in our case) with predefined logic and access to specialised tools (e.g. Pandas data analysis library) to allow natural language queries for data analysis.
5. Pipeline reproducer: extracts executed function calls and code from a chat history, organising them into a new Python function that can be reused in the future. Its system prompt instructs it to extract only the functions and code snippets that were actually executed, while excluding the failed ones. The system prompt also provides an example of an extracted pipeline to ensure the output is valid and can be re-run on new datasets without modification.
6. RAG agent: expands PhenoAssistant’s domain-specific knowledge by embedding and retrieving information from literature related to  user-specified tasks.

### Reporting summary

Further information on research design is available in the Nature Portfolio Reporting Summary linked to this article.

## Supplementary information

Supplementary Information

Peer Review File

Reporting Summary

## Source data

Source Data

## Data Availability

The *Arabidopsis thaliana* data used in case study 1 have been deposited in the Zenodo repository [10.5281/zenodo.18940282]^76^. The data used for training and evaluating the computer vision model used in case study 1 are publicly available from the CVPPP2017 Leaf Segmentation Challenge dataset (A1 and A4 subsets) at CodaLab [https://codalab.lisn.upsaclay.fr/competitions/8970]. The potato data used in case study 2 are publicly available in the Zenodo repository [10.5281/zenodo.7938231]^77^. The winter wheat data used in case study 3 are publicly available from the CVPPA@ICCV'23: image classification of nutrient deficiencies in winter wheat and winter rye dataset (WW2020 subset) at CodaLab [https://codalab.lisn.upsaclay.fr/competitions/13833]. Source data are provided with this paper.

## Code availability

The code for this research, as well as the chat logs and generated outputs of the case studies and evaluations, are available at Github [https://github.com/vios-s/PhenoAssistant/]^78^.
