# Supplementary material for: A conversational multi-agent AI system for automated plant phenotyping
Source: Nat Commun. 2026 Apr 3;17:6391. doi: 10.1038/s41467-026-71090-y (PMC13376813; doi:10.1038/s41467-026-71090-y)
Supplement: Supplementary file 1 — Supplementary Information [file 41467_2026_71090_MOESM1_ESM.pdf]

A conversational multi-agent AI system for automated plant phenotyping

Chen *et al.*

Supplementary Note 1. Chat log for Case Study 1 task 1

Here, we provide the user's prompt and PhenoAssistant's response of Case Study 1 Task 1 to illustrate how PhenoAssistant operates behind the scene. Similar processes apply to other tasks.

User's prompt

```
I have an image dataset consisting of 24 Arabidopsis plants associated with
different ecotypes. Each plant has multiple images taken at different days
after sowing.

The metadata for the dataset is stored in ./data/aracrop_metadata.json. The
metadata contains the following keys for each image: file_name, plant_id,
ecotype, and days_after_sowing.

Your task is to compute the following phenotypes for each image, the pixel to
cm mapping scale is 0.03:
1. leaf count
2. projected leaf area (PLA)
3. average leaf area
4. diameter of the whole plant
5. perimeter of the whole plant
6. compactness of the whole plant
7. stokinness of the whole plant

Merge the computed phenotypes with the metadata information and save them in
./results/Case1/aracrop_phenotypes.csv. Make sure the file names match.
```

PhenoAssistant's response

To accomplish this task, we will follow these steps:

Instance Segmentation: Perform instance segmentation on the images to identify individual leaves and the whole plant.

Compute Phenotypes: Calculate the required phenotypes from the instance segmentation results.

Merge Metadata: Merge the computed phenotypes with the metadata information.

Save Results: Save the merged data to a CSV file.

Let's proceed step-by-step:

Step 1: Instance Segmentation

First, we need to check the available checkpoints for instance segmentation and select a suitable one.

Step 2: Compute Phenotypes

Once we have the instance segmentation results, we will compute the phenotypes using the specified pixel-to-cm mapping scale.

Step 3: Merge Metadata

We will merge the computed phenotypes with the metadata information.

Step 4: Save Results

Finally, we will save the merged data to the specified CSV file.

Let's start by checking the available checkpoints for instance segmentation.

```
get_model_zoo()
```

We have the following checkpoints available for instance segmentation:

arabidopsis_leaf-instance-segmentation_cvppp2017-a1a4_m2fb_fullft

potato_leaf-instance-segmentation_leaf-only-sam

...

Since our dataset consists of Arabidopsis plants, we will use the arabidopsis_leaf-instance-segmentation_cvppp2017-a1a4_m2fb_fullft checkpoint.

Let's start with the **instance segmentation**.

```
infer_instance_segmentation(
    file_path = './data/aracrop_metadata.json',
    checkpoint = 'arabidopsis_leaf-instance-segmentation_cvppp2017-a1a4_m2fb_fullft',
    output_dir = './results/Case1'
)
```

The instance segmentation results are **saved at** ./results/Case1/ins_seg_results.json using COCO format.

Let's proceed with **computing phenotypes**.

```
compute_phenotypes_from_ins_seg(
    ins_seg_result_path = './results/Case1/ins_seg_results.json',
    save_path = './results/Case1/phenotypes.csv',
    pixel_to_cm = 0.03
)
```

The computed phenotypes **are saved to ./results/Case1/phenotypes.csv** as a list of dictionaries, each containing the following keys: 'file_name', 'leaf_count', 'average_leaf_area', 'projected_leaf_area', 'diameter', 'perimeter', 'compactness', 'stockiness'.

Let's proceed with **merging metadata and saving results**.

```
coding("message" = "Merge the computed phenotypes from ./results/Case1/phenotypes.csv
with the metadata information from ./data/aracrop_metadata.json and save the result
to ./results/Case1/aracrop_phenotypes.csv. Ensure the file names match.")
```

Code generation and execution:

```
import pandas as pd
import json

# Load the phenotypes data
phenotypes_df = pd.read_csv('./results/Case1/phenotypes.csv')

# Load the metadata
with open('./data/aracrop_metadata.json', 'r') as file:
    metadata = json.load(file)

# Convert metadata to DataFrame
metadata_df = pd.DataFrame(metadata)

# Merge the phenotypes with metadata using 'file_name' as the common key
merged_df = pd.merge(phenotypes_df, metadata_df, on='file_name')

# Save the merged DataFrame to a new CSV file
merged_df.to_csv('./results/Case1/aracrop_phenotypes.csv', index=False)
```

The task involved merging the computed phenotypes from ./results/Case1/phenotypes.csv with the metadata information from ./data/aracrop_metadata.json and saving the result to ./results/Case1/aracrop_phenotypes.csv.

The necessary steps were:

Load the phenotypes data from ./results/Case1/phenotypes.csv.

Load the metadata from ./data/aracrop_metadata.json.

Convert the metadata to a DataFrame.

Merge the phenotypes DataFrame with the metadata DataFrame using the common key file_name.

Save the merged DataFrame to ./results/Case1/aracrop_phenotypes.csv.

The final merged file has been successfully saved to: ./results/Case1/aracrop_phenotypes.csv

Task Summary: The phenotypes have been successfully computed and merged with the metadata information. The final results are saved in ./results/Case1/aracrop_phenotypes.csv.

Supplementary Note 2. Pipeline reproduction

Based on the conversation from Case Study 1 Task 1, we can prompt PhenoAssistant to save the executed tools and code as a reusable pipeline. To achieve this, PhenoAssistant invokes the Pipeline Reproducer agent to extract the relevant components and save them in a Python function. An example of the user prompt and the corresponding extracted pipeline is provided at the end of this note.

To evaluate this functionality, we selected four representative tasks. For each task, we instructed PhenoAssistant to extract the pipeline five times. The extracted pipelines for each task were then applied to the same input file, and we assessed whether they could be executed successfully and produce consistent and correct results. The evaluated tasks and the key tool calls involved are:

1. Case Study 1 Task 1 (Extract and Save Arabidopsis Phenotypes):

   Instance Segmentation Module → Phenotype Computation Module → Code Writer Agent

2. Case Study 1 Task 2 (Compute and Plot Statistics of Arabidopsis Phenotypes):

   Table Analyser Agent → Data Visualiser Agent

3. Case Study 1 Task 4 (Perform Statistical Tests on Arabidopsis Phenotypes):

   Statistical Test Module (ANOVA) → Statistical Test Module (Tukey-Kramer)

4. Case Study 2 Task 1 (Extract and Save Potato Phenotypes):

   Instance Segmentation Module → Phenotype Computation Module → Code Writer Agent

These tasks span two plant species and different phenotype analysis purposes. They also represent diverse pipeline structures, including mixed module–agent sequences (Case Study 1 Task 1, Case Study 2 Task 1), pure agent-based sequences (Case Study 1 Task 2), and pure module-based sequences (Case Study 1 Task 4).

We observed that the extracted pipelines (Python functions) varied in content and structure across the five runs for each task, reflecting inherent LLM stochasticity. Despite these variations, all pipelines, except those from Case Study 1 Task 2, were executed successfully and produced consistent, correct results within each task. For Case Study 1 Task 2, one run failed because the extracted pipeline was not executable, while the remaining runs produced correct results. These results demonstrate the generalisability and robustness of PhenoAssistant's pipeline reproduction capability. Below we show an example of the user's prompt along with the extracted and saved pipeline in Case Study 1 Task 1.

User's prompt

```
Summarise the executed function calls and code into a reproducible pipeline.
Name it as ara_crop_pipeline.
```

Extracted and saved pipeline

```
def ara_crop_pipeline(metadata_path: Annotated[str, 'Path to the metadata
JSON file'], output_dir: Annotated[str, 'Directory to save the results'],
pixel_to_cm: Annotated[float, 'Pixel to cm mapping scale'] = 0.03) ->
Dict[str, Any]:
    """
    Pipeline to compute phenotypes for Arabidopsis plant images, merge with
metadata, and save the results.
    Args:
        metadata_path (str): Path to the metadata JSON file.
        output_dir (str): Directory to save the results.
        pixel_to_cm (float): Pixel to cm mapping scale. Default is 0.03.
    Returns:
        Dict[str, Any]: Status and path to the saved results.
    """

    import json
    import pandas as pd

    try:
        logging.info("Starting ara_crop_pipeline execution.")

        # Step 1: Check available checkpoints for instance segmentation
```

```
        logging.info("Checking available checkpoints for instance
segmentation.")
        checkpoints = get_model_zoo()
        selected_checkpoint = "fengchen025/arabidopsis_leaf-instance-
segmentation_cvppp2017-a1a4_m2fb_fullft"

        # Step 2: Perform instance segmentation
        logging.info("Performing instance segmentation.")
        infer_instance_segmentation(file_path=metadata_path,
checkpoint=selected_checkpoint, output_dir=output_dir)

        # Step 3: Compute phenotypes from instance segmentation results
        logging.info("Computing phenotypes from instance segmentation
results.")
        ins_seg_result_path = f"{output_dir}/ins_seg_results.json"
        phenotypes_save_path = f"{output_dir}/phenotypes.csv"

compute_phenotypes_from_ins_seg(ins_seg_result_path=ins_seg_result_path,
save_path=phenotypes_save_path, pixel_to_cm=pixel_to_cm)

        # Step 4: Merge computed phenotypes with metadata
        logging.info("Merging computed phenotypes with metadata.")
        phenotypes_df = pd.read_csv(phenotypes_save_path)
        with open(metadata_path, 'r') as file:
            metadata = json.load(file)
        metadata_df = pd.DataFrame(metadata)
        merged_df = pd.merge(phenotypes_df, metadata_df, on='file_name')
        final_save_path = f"{output_dir}/aracrop_phenotypes.csv"
        merged_df.to_csv(final_save_path, index=False)

        logging.info("Pipeline executed successfully.")
        return {"status": "success", "result_path": final_save_path}

    except Exception as e:
        logging.error(f"Pipeline execution failed: {str(e)}", exc_info=True)
        return {"status": "error", "message": str(e)}
```

Supplementary Note 3. Details and prompts of the evaluation for tool selection

The aim of this evaluation is to assess whether PhenoAssistant can generate the correct pipeline by chaining the integrated tools for a plant phenotyping task requiring multiple steps (e.g. using computer vision models and generated code). The focus is on the pipeline, not the final outputs, as they rely on the tools' accuracy. Consequently, some of the following tasks are synthetic (i.e. only descriptions are provided without the actual input data) since accessing their results is unnecessary. The complete evaluation results are presented at Supplementary Table 2. The prefix prompt and the prompt for each task sent to PhenoAssistant are shown below.

Prefix prompt

Each task prompt starts with:

```
Output the tools and their arguments in the correct order in JSON format to perform the following task.
Do not attempt to execute any tools. Do not explain anything.
Here is the task:
```

Task 1: Linear regression on leaf morphology

```
You have a CSV file at ./data/leaf_morphology.csv containing 50 records with columns: file_name, leaf_diameter, and leaf_weight.
First, clean the data by removing any records with missing values.
Next, fit a linear regression with leaf_diameter as x and leaf_weight as y.
Report the equation of the regression line, the R^2 value, and generate a plot of the regression at ./results/leaf_length_vs_width.png.
```

Task 2: Merging experiments and ANOVA analysis

```
You have several CSV files in ./data/exp_results/ representing different experimental replicates.
Merge these files on the column 'sample_id'.
After merging, perform an ANOVA test on the column 'chlorophyll_content' across different treatment groups.
Output the ANOVA summary table as ./results/anova_chlorophyll.csv.
```

Task 3: Area measurement in flower images

You have a folder of images at ./images/flower_plants/ and a metadata CSV at ./data/flower_metadata.csv with columns: file_name and bloom_status.

For each image, measure its area.

Merge these area measurements with the metadata, and then create a boxplot comparing area distributions for different bloom_status values.

Save the boxplot as ./results/flower_area_boxplot.png.

Task 4: Wheat spike phenotype extraction

Using the metadata file './data/wheat_metadata.json' (which contains keys: file_name, variety, and acquisition_date), extract the phenotypes {spike count, spike length} for each wheat image. Merge these phenotypes with themetadata and save the combined data to './results/wheat_phenotypes.csv'.

Task 5: Wheat variety spike count analysis

Given './results/wheat_phenotypes.csv', perform a statistical analysis to test whether there is a significant difference in spike count between different wheat varieties.

Use an appropriate statistical test and output the test statistics and p-value to a text file.

Task 6: Wheat growth phenotyping

You have a set of wheat images with a metadata file './data/wheat_metadata.csv' (with columns: file_name, plant_id, date, treatment).

Compute leaf count and leaf area for each image.

Merge the computed phenotypes with the metadata and save to './results/wheat_growth_phenotypes.csv', then generate comparative plots of leaf count and area by treatment group.

Summarise these plots for different treatments.

Task 7: Stress prediction

Predict stress levels from images listed in the metadata file './data/stress_metadata.csv' (with columns: file_name, manual_stress_level).

Compare these predictions against manually scored stress levels provided in the metadata using confusion matrix. Plot the confusion matrix and save it as './results/stress_confusion_matrix.png'.

Task 8: Maximum leaf count extraction

Given './data/arabidopsis_phenotypes.csv', determine which ecotype has the maximum leaf count at day 26.

Generate a bar plot showing the maximum leaf count for each ecotype at day 26.

Task 9: Extracting and summarising field observations

You have a file at ./data/field_observations.csv containing plant phenotyping observations with various attributes. Extract all records where the attribute'disease_symptom' is true, and generate a summary plot counting the frequency of each symptom type.

Task 10: Maize root phenotyping and yield correlation

Given a dataset of maize root images stored in './data/maize_roots/' and metadata in './data/maize_metadata.csv' (which includes file_name, plant_id, and yield).

Extract the total root length for each image.

Find the correlation coefficient between the root length and yield.

Task 11: Soybean pod counting

You have a CSV metadata at ./data/soybean_metadata.csv containing 200 records with keys "file_name" (image names), "plant_id", and "planting_date".

You task is to extract the number of soybean pods per image.

Merge these results with the existing metadata by adding a new column named pod_count. Save the merged file to ./results/soybean_pod_counts.csv.

Task 12: Training a drought stress identification model

I have some surghum leaf images in ./images/sorghum_leaf/ and metadata in ./data/sorghum_leaf_metadata.csv (columns: file_name, treatment, drought_stress).

I would like to build a model that can automatically identify the drought stress given a surghum leaf image. Can you tell me what to do?

Task 13: Calculate and plot leaf area/counting ratio

You have a CSV file ./data/phenotypes.csv with columns: plant_id, leaf_area, leaf_count.

Compute the ratio of leaf_area / leaf_count for each plant and save as ./results/leaf_area_count_ratio.csv.

Generate a histogram of the ratio values across all plants and save the plot as ./results/leaf_area_ratio_count_hist.png.

Task 14: Rice growth stage summary

You have a CSV file at ./data/rice_growth_stages.csv with columns: plant_id, day_after_sowing, growth_stage ("vegetative" or "reproductive").

Plot the number of plants in each growth stage over days after sowing, save the plot at ./results/rice_stage_counts.png, and provide a summary of the plot.

Task 15: Fruit size extraction and growth curve plot

You have some fruit images with the metadata at ./data/tomato_metadata.json with keys: file_name, plant_id, day_after_flowering.

Your task is to extract the fruit diameter for each image, save the results at ./results/fruit_diameters.csv.

Also plot the mean fruit diameter over time (day_after_flowering) across all plants. Save the plot at ./results/fruit_growth.png.

Task 16: Pipeline reproduction on rice trait analysis

You have a CSV ./data/rice_traits.csv with columns: plant_id, day, leaf_area, treatment.

You need to complete the following tasks:

(1) Compute and plot the mean leaf_area vs. day for each treatment group. Save the plot to ./results/rice_leaf_growth.png.

(2) Save this workflow as a reusable pipeline named rice_trait_analysis.

(3) Apply the pipeline to ./data/rice_traits_new.csv.

Task 17: Chlorophyll index calculation and plotting

Given a CSV file at ./data/leaf_reflectance.csv with columns: plant_id, red, and nir. Red and nir represent reflectance values in the visible red band and near-infrared band, respectively.

Compute the NDVI for each plant using (nir - red) / (nir + red) and merge the results with the original CSV by adding a new column named ndvi. Save the merged file to ./results/leaf_ndvi.csv.

Also plot the NDVI for each plant on a bar chart and save it to './results/ndvi_chart.png'.

Task 18: Wheat biomass over treatments

You have a CSV ./data/wheat_biomass.csv with columns: plant_id, treatment, biomass.

Compute and plot mean biomass for each treatment group with error bars and save as ./results/wheat_biomass_plot.png.

Analyse the pattern of the plot to determine whether some treatments consistently produce higher biomass than others.

Task 19: Sunflower head diameter growth trend

You have a CSV ./data/sunflower_heads.csv with columns: plant_id, head_diameter, day.

Compute and plot the mean head diameter over days save as ./results/sunflower_head_growth.png.

Analyse the plotted trend and determine whether head diameter growth appears a linear or an exponential pattern.

Task 20: Tomato ripening identification

You have some tomato fruit images with metadata ./data/tomato_metadata.csv (column: file_name).

Classify each fruit into ripening stages and create a bar chart showing the frequency of each class.

Save the chart as ./results/tomato_ripening_stages.png.

Supplementary Note 4. Details and pompts of the evaluation for vision model selection

Vision Model Selection I

The goal of this evaluation is to assess if PhenoAssistant is able to recommend the appropriate type of computer vision model (from instance segmentation, image classification, and image regression), for a given plant phenotyping task. The prompt sent to PhenoAssistant is:

```
For the following plant phenotyping tasks, what models will you recommend me
to train? Choose from instance segmentation, image classification, and image
regression.

1. Leaf counting
2. Are these plants healthy or diseased?
3. Leaf area estimation
4. Plant species identification
5. Flower counting
6. Fruit counting
7. Root tip localisation
8. Determine the type of leaf shape in each image
9. Plant height estimation
10. Which growth stage are these plants in, vegetative or flowering?
11. Plant biomass estimation
12. What type of nutrient deficiency is present in these plants?
13. Leaf angle measurement
14. Highlight the weeds in these field images
15. Stem counting in multi-stem plants
16. Describe the severity of disease symptoms in these plants (mild,
moderate, or severe)
17. Fruit size estimation
18. Identify the colour of flowers in these images
19. Does these plants appear abiotic or biotic stress?
20. Herbivory damage estimation (chewed leaf area)
21. Root length estimation
22. What type of seeds is in this image?
23. Are the shapes of these flowers similar or different?
24. Does these image contain visible seedling emergence?
25. Assess the greenness of these leaves
26. Tiller counting in cereals
27. Pod counting in legumes
```

28. For each image, tell me which riping stage the fruits are in
29. Berry maturity score
30. Delineate the crop rows in these images
31. Leaf chlorophyll content estimation
32. Are these fruits ripe or unripe?
33. Root nodule counting
34. Are the plants in these images standing upright or have they fallen over?
35. Quantify the severity of flower diseases in a continuous scale
36. Calculate the affected area of the leaf disease in these images
37. Measure the length of root hairs in these images
38. Estimate the photosynthetic efficiency of these plants using Normalised Difference Vegetation Index (NDVI)
39. Identify the stress level of these plants (mild or severe)
40. Fruit cracking severity assessment on a discrete scale of 1 (no cracking) to 5 (severe cracking)
41. What is the maturity status of these pods?
42. Bud counting
43. Identify the type of insect pest damage on leaves
44. Plant density estimation
45. What branching pattern best describes the roots in these images?
46. Plant vigor scoring on a continuous scale of 0 to 1
47. Assess the level of thrips damage on flowers
48. Identify the type of shape of the leaf margins in these images
49. Vine length estimation
50. Grape cluster counting

PhenoAssistant's matches are presented below, and all are correct:

1. Leaf counting: Instance segmentation
2. Are these plants healthy or diseased?: Image classification
3. Leaf area estimation: Image regression
4. Plant species identification: Image classification
5. Flower counting: Instance segmentation
6. Fruit counting: Instance segmentation
7. Root tip localisation: Instance segmentation
8. Determine the type of leaf shape in each image: Image classification
9. Plant height estimation: Image regression
10. Which growth stage are these plants in, vegetative or flowering?: Image classification
11. Plant biomass estimation: Image regression
12. What type of nutrient deficiency is present in these plants?: Image classification

13. Leaf angle measurement: Image regression
14. Highlight the weeds in these field images: Instance segmentation
15. Stem counting in multi-stem plants: Instance segmentation
16. Describe the severity of disease symptoms in these plants (mild, moderate, or severe): Image classification
17. Fruit size estimation: Image regression
18. Identify the colour of flowers in these images: Image classification
19. Does these plants appear abiotic or biotic stress?: Image classification
20. Herbivory damage estimation (chewed leaf area): Image regression
21. Root length estimation: Image regression
22. What type of seeds is in this image?: Image classification
23. Are the shapes of these flowers similar or different?: Image classification
24. Does these image contain visible seedling emergence?: Image classification
25. Assess the greenness of these leaves: Image regression
26. Tiller counting in cereals: Instance segmentation
27. Pod counting in legumes: Instance segmentation
28. For each image, tell me which riping stage the fruits are in: Image classification
29. Berry maturity score: Image regression
30. Delineate the crop rows in these images: Instance segmentation
31. Leaf chlorophyll content estimation: Image regression
32. Are these fruits ripe or unripe?: Image classification
33. Root nodule counting: Instance segmentation
34. Are the plants in these images standing upright or have they fallen over?: Image classification
35. Quantify the severity of flower diseases in a continuous scale: Image regression
36. Calculate the affected area of the leaf disease in these images: Image regression
37. Measure the length of root hairs in these images: Image regression
38. Estimate the photosynthetic efficiency of these plants using Normalised Difference Vegetation Index (NDVI): Image regression
39. Identify the stress level of these plants (mild or severe): Image classification
40. Fruit cracking severity assessment on a discrete scale of 1 (no cracking) to 5 (severe cracking): Image classification
41. What is the maturity status of these pods?: Image classification
42. Bud counting: Instance segmentation
43. Identify the type of insect pest damage on leaves: Image classification
44. Plant density estimation: Image regression
45. What branching pattern best describes the roots in these images?: Image classification

```
46. Plant vigor scoring on a continuous scale of 0 to 1: Image regression
47. Assess the level of thrips damage on flowers: Image classification
48. Identify the type of shape of the leaf margins in these images: Image
classification
49. Vine length estimation: Image regression
50. Grape cluster counting: Instance segmentation
```

Vision model selection II

The goal of this evaluation is to assess whether PhenoAssistant can identify the most suitable specific model from a vision model zoo for a given plant phenotyping task. The prompt sent to PhenoAssistant is:

```
For the following plant phenotyping tasks, choose the most suitable model
from the model zoo that can effectively address each task.

For each plant phenotyping task, output your answer as "task: selected vision
model"

Plant phenotyping tasks:
['Delineate the crop rows in these soybean field images.',
'Do these tomato plants appear under abiotic or biotic stress?',
'Identify all the wheat spikes in these images.',
'How mature these soybean pods appear?',
'Infer chlorophyll concentration of these spinach leaves.',
'Score the maturity of blueberries presented in these images.',
'Can you compute the leaf area of these Arabidopsis plants?',
'Highlight all the clusters in these grape images.',
'Recognise and record the species of these wildflowers.',
'How badly are these sunflowers affected by disease? Provide a score for each
image.',
'Pod counting for peas.',
'Quantify structural traits related to vine extension in these cucumber
images.',
'Determine the ripening progress of these tomato fruits.',
'Rank the maize individuals by vigour on a continuous scale.',
'Determine the stress level (mild or severe) of the given rice plants.',
'Map non-crop vegetation appearing in these wheat field photographs.',
'Can you estimate the leaf greenness of these lettuces?',
'Compute the above-ground biomass for these barley images.',
'Assess whether these potato plants appear healthy or illed.',
'What type of pest damage can be found in these cabbage leaves?']
```

```
Model zoo:
{'instance-segmentation':
    ['wildflower_cluster-instance-segmentation_m2fb',
    'cabbage_leaf-pest-instance-segmentation_m2fb',
    'wheat_spike-instance-segmentation_m2fb',
    'wheat_weed-instance-segmentation_m2fb',
    'tomato_fruit-instance-segmentation_m2fb',
    'wheat_leaf-instance-segmentation_m2fb',
    'arabidopsis_leaf-instance-segmentation_m2fb',
    'soybean_pod-instance-segmentation_m2fb',
    'grape_cluster-instance-segmentation_m2fb',
    'wheat_stem-instance-segmentation_m2fb'],
'image-classification':
    ['cucumber_disease-classify_dino2b',
    'maize_vigour-classify_dino2b',
    'cabbage_leaf-pest-damage-classify_dino2b',
    'potato_health-disease-classify_dino2b',
    'rice_stress-level-classify_dino2b',
    'sunflower_disease-classify_dino2b',
    'tomato_fruit-ripening-stage-classify_dino2b',
    'soybean_pod-maturity-status-classify_dino2b',
    'wildflower_species-classify_dino2b',
    'rice_disease-classify_dino2b'],
'image-regression':
    ['grape_cluster-number-regress_dino2b',
    'arabidopsis_leaf-greenness-regress_dino2b',
    'rice_biomass-regress_dino2b',
    'maize_vigour-regress_dino2b',
    'lettuce_leaf-greenness-regress_dino2b',
    'cucumber_vine-length-regress_dino2b',
    'sunflower_disease-severity-regress_dino2b',
    'arabidopsis_disease-severity-regress_dino2b',
    'potato_disease-severity-regress_dino2b',
    'barley_biomass-regress_dino2b']}
```

PhenoAssistant's task–model matching results are shown below. All matches are correct. For tasks without a suitable model in the model zoo, PhenoAssistant suggests an appropriate model type for fine-tuning.

1. Delineate the crop rows in these soybean field images: No specific model available. Suggest finetuning a new instance segmentation model.
2. Do these tomato plants appear under abiotic or biotic stress?: No specific model available. Suggest finetuning a new image classification model.
3. Identify all the wheat spikes in these images: wheat_spike-instance-segmentation_m2fb
4. How mature these soybean pods appear?: soybean_pod-maturity-status-classify_dino2b
5. Infer chlorophyll concentration of these spinach leaves: No specific model available. Suggest finetuning a new image regression model.
6. Score the maturity of blueberries presented in these images: No specific model available. Suggest finetuning a new image classification model.
7. Can you compute the leaf area of these Arabidopsis plants?: arabidopsis_leaf-instance-segmentation_m2fb
8. Highlight all the clusters in these grape images: grape_cluster-instance-segmentation_m2fb
9. Recognise and record the species of these wildflowers: wildflower_species-classify_dino2b
10. How badly are these sunflowers affected by disease? Provide a score for each image: sunflower_disease-severity-regress_dino2b
11. Pod counting for peas: No specific model available. Suggest finetuning a new instance segmentation model.
12. Quantify structural traits related to vine extension in these cucumber images: cucumber_vine-length-regress_dino2b
13. Determine the ripening progress of these tomato fruits: tomato_fruit-ripening-stage-classify_dino2b
14. Rank the maize individuals by vigour on a continuous scale: maize_vigour-regress_dino2b
15. Determine the stress level (mild or severe) of the given rice plants: rice_stress-level-classify_dino2b
16. Map non-crop vegetation appearing in these wheat field photographs: wheat_weed-instance-segmentation_m2fb
17. Can you estimate the leaf greenness of these lettuces?: lettuce_leaf-greenness-regress_dino2b
18. Compute the above-ground biomass for these barley images: barley_biomass-regress_dino2b
19. Assess whether these potato plants appear healthy or illed: potato_health-disease-classify_dino2b
20. What type of pest damage can be found in these cabbage leaves?: cabbage_leaf-pest-damage-classify_dino2b

Supplementary Note 5. Details and prompts of the evaluation for data analysis

The goal of this evaluation is to evaluate whether PhenoAssistant can produce the correct outputs for data analysis requests including extracting values from CSV files, computing statistics, generating plots, analysing plots, converting file formats, computing and analysing new metrics). This assesses the reliability of the built-in LLM agents (e.g. table analyser and data visualiser) for data analysis.

The task prompts sent to PhenoAssistant for each task are shown below. For Tasks 11, 12, and 14, PhenoAssistant generates incorrect responses; hence, both the incorrect outputs and the corresponding correct answers are included.

Task 1: Extracting values

```
Given ./data_for_eval/aracrop_phenotypes.csv, determine the maximum leaf
count and the corresponding plant id at day 26.
```

Task 2: Extracting values

```
Given ./data_for_eval/aracrop_phenotypes.csv, determine the minimum leaf
count and the corresponding plant id at day 1.
```

Task 3: Extracting values

```
Given ./data_for_eval/potato_metadata.csv, find the maximum manually measured
dried weight in variety Voyager and the corresponding file name.
```

Task 4: Extracting values

```
Given ./data_for_eval/potato_metadata.csv, find the minimum manually measured
dried weight in variety Voyager and the corresponding file name.
```

Task 5: Computing statistics

```
Given ./data_for_eval/aracrop_phenotypes.csv, compute the mean and standard
deviation of projected leaf area for ecotype ein2 on day 26.
```

Task 6: Computing statistics

```
Given ./data_for_eval/potato_metadata.csv, compute the mean and standard
deviation of manually measured leaf area of Desiree.
```

Task 7: Generating plots

```
Given ./data_for_eval/aracrop_phenotypes.csv, plot the leaf count for plant 1
against days. Save the results at ./results_for_eval/task7.png.
```

Task 8: Generating plots

```
Given ./data_for_eval/aracrop_phenotypes.csv, create a bar chart of leaf
count for every plant at day 26. Save the plot
at ./results_for_eval/task8.png.
```

Task 9: Generating plots

```
Given ./data_for_eval/potato_metadata.csv, use bar charts to plot the
manually measured dried weight for every plant. Save the plot
at ./results_for_eval/task9.png.
```

Task 10: Generating plots

```
Given ./data_for_eval/potato_metadata.csv, create a histogram for manually
measured leaf area for every plant. Save the plot
at ./results_for_eval/task10.png.
```

Task 11: Analysing plots

```
Given ./results_for_eval/task7.png, at which day does the leaf count first
reach 14?
```

Incorrect response provided by PhenoAssistant: day 19.

Correct answer: day 15.

Task 12: Analysing plots

```
Given ./results_for_eval/task8.png, which plant has the maximum leaf count at
day 26?
```

Incorrect response provided by PhenoAssistant: plant 6.

Correct answer: plant 5.

Task 13: Analysing plots

```
Given ./results_for_eval/task9.png, are most plants have a dried weight less
than 0.8?
```

Task 14: Analysing plots

```
Given ./results_for_eval/task10.png, what is the most frequent range of leaf
area values?
```

Incorrect response provided by PhenoAssistant: between 0 and 100 with a frequency of 14.

Correct answer: between 0 and 33 with a frequency of 14 (or between 0 and 100 with a frequency of 18).

Task 15: Converting file formats

```
Save './data_for_eval/potato_metadata.csv' to a JSON file
at ./results_for_eval/potato_metadata.json.
```

Task 16: Converting file formats

```
Save ./results_for_eval/potato_metadata.json as a csv file but only keep the
columns of "file_name", "Variety", and "manual_leaf_area". Save it
to ./results_for_eval/potato_metadata_subset.csv.
```

Task 17: Computing statistics

```
Given ./data_for_eval/potato_metadata.csv, compute the mean and standard
deviation of manually measured dried weight of Voyager.
```

Task 18: Computing statistics

```
Given ./data_for_eval/aracrop_phenotypes.csv, compute the mean and standard
deviation of leaf count for ecotype pgm at day 1.
```

Task 19: Computing new metrics

```
Given ./data_for_eval/potato_metadata.csv, compute the leaf area to dried
weight ratio using this formula: manual_leaf_area/manual_dried_weight for
each file.
Save the results to ./results_for_eval/potato_ratio.csv.
```

Task 20: Analysing new metrics

Given ./results_for_eval/potato_ratio.csv, tell me which Variety has the higher average leaf area to dried weight ratio.

Supplementary Note 6. System prompts

This section presents the system prompts used to configure various LLM agents integrated into PhenoAssistant, each tailored for specific functionalities. The system prompts of the Tool Evaluator and the Critic agent used in tool selection evaluation are also provided.

Manager

```
manager. Use the available tools to solve plant-related tasks.

Instructions:
1. Begin by creating a clear, step-by-step plan for the task. Provide an
explanation of each step before proceeding. You may refine the plan as needed
based on intermediate results.
```

2. When using multiple tools, be sure to provide the outputs from one tool to the next as inputs if necessary.

3. When you need to use coding-related tools, provide clear descriptions of the task requirements and expected outputs. Do not pass concrete codes to the coding related functions.

4. When you need to use computer vision to solve a task, first check the available checkpoints using the get_model_zoo function:

    - Select a suitable checkpoint for the task if it it exists. Make sure you pass a list of image paths (e.g. [./data/image1.png, ./data/image2.png, ...]), or a single csv/json file (e.g. ./data/metadata.json) to the corresponding inference function.

    - If no suitable checkpoint is available, suggest user to finetune a new model. DO NOT rely on online source. Use the get_dataset_format function to instruct the user to provide a training dataset.

5. When a user ask you to execute a saved pipeline, first call get_pipeline_zoo to know what pipelines are available, and then call get_pipeline_info} to understand how to use the selected pipeline.

6. At the end of the task, provide a summary of the results and ask the user if they need any further assistance.

Return "TERMINATE" when the task is completed.

Code writer

code_writer. Write python codes to accomplish a given task.

General instructions:

    1. Write the full Python code solution in a single code block. Do not return incomplete codes or multiple code blocks.

    2. code_executor will execute the code. If the output shows errors, correct them and present the complete, updated code.

    3. Wait until the code_executor completes the execution and returns the output. If the output shows task completion, return a single 'TERMINATE'. Do not write or print it within the code block.

Data visualiser

code_writer. Write python codes to accomplish a given task.

General instructions:

    1. Write the full Python code solution in a single code block. Do not return incomplete codes or multiple code blocks.

2. code_executor will execute the code. If the output shows errors, correct them and present the complete, updated code.

3. Wait until the code_executor completes the execution and returns the output. If the output shows task completion, return a single 'TERMINATE'.

If you are asked to plot data, follow the guidance below:

- Carefully follow the user's requirements of the expected figure.
- Include clear and descriptive titles, axis labels, and legends in the figure.
- Ensure the figure is visually appealing and easy to read by adjusting its styling elements such as font sizes, line widths, and colors.

Preferred Python libraries for plotting:

- matplotlib, seaborn, plotly

Plot analyser

plot_analyser. You are a plant data scientist to analyse figures.

Pipeline reproducer

pipeline_summariser. Extract executed function calls and Python code snippets from a chat log, in order to form a pipeline that can be reused in the future.

Instructions:

- Output the pipeline as a single Python function with key arguments.
- Provide a clear description, annotate all key arguments with their data types and descriptions, and specify the output type.
- Include only the executed function calls and Python code snippets. Do not include the unexcuted ones.
- For code snippets, include them exactly as they were executed, including the library imports.
- For function calls, you don't need to import them, just include the function execution with the same parameters that were used.

Here is an example of the expected output:

```
def pipeline_name(param1: Annotated[str, 'description of param1']
                        = default_value_param1,
                  param2: Annotated[int, 'description of param2']
                        = default_value_param2) -> Dict[str, Any]:
    """
    Pipeline description.
    Returns:
        Dict[str, Any]: description of the output.
```

```
    """

    import necessary_libraries  # Dynamically extract required imports

    try:
        logging.info("Starting pipeline execution.")

        # Step 1: Call function A
        logging.info("Executing function_call_1")
        result_1 = function_call_1(param1)

        # Step 2: Execute Python code snippet
        logging.info("Executing extracted Python logic for something.")
        def some_python_logic(input_data):
            """ Extracted Python code block. """
            # { Insert copied code blocks here }
            return output_data

        result_2 = some_python_logic(result_1)

        # Step 3: Call function B
        logging.info("Executing function_B")
        final_result = function_B(result_2, param2)

        logging.info("Pipeline executed successfully.")
        return {"status": "success", "result": final_result}

    except Exception as e:
        logging.error(f"Pipeline execution failed: {str(e)}",
                      exc_info=True)
        return {"status": "error", "message": str(e)}
```

Tool evaluator

```
tool evaluator. Given a user provided task, you need to evaluate the chain of
tools generated by an agent. Here are the available tools to the agent (name
→ description, parameters):
{available_tools_str}

Instructions:
```

1. First, check whether the overall chain makes sense to address the task, whether any important steps are missing, and whether the tools are used in a correct order.
2. Then, check each step:
    - Verify the tool exists. Make sure the tool name is correct.
    - Verify the tool is appropriate for the step. Make sure the tool usage fits the tool description.
    - Verify the provided arguments to the tool. Make sure they align with the task description provided by the user and the tool's parameter schema.

Output four scores (ranged 1-5) for each of the above checks, with 1 being the worst and 5 being the best:
    - Overall chain correctness (1-5)
    - Tool existence correctness (1-5)
    - Tool appropriateness correctness (1-5)
    - Argument correctness (1-5)}

Critic

critic. Given a user provided task, evaluate and suggest corrections to refine the chain of tools generated by the manager. Here are the available tools to the manager (name → description, parameters):
{available_tools_str}

Instructions:
1. First, check whether the overall chain makes sense to address the task, whether any important steps are missing, and whether the tools are used in a correct order.
2. Then, check each step:
    - Verify the tool exists. Make sure the tool name is correct.
    - Verify the tool is appropriate for the step. Make sure the tool usage fits the tool description.
    - Verify the provided arguments to the tool. Make sure they align with the task description provided by the user and the tool's parameter schema.

If anything needs to be corrected, clearly point out the issues found, be specific about the step and argument.

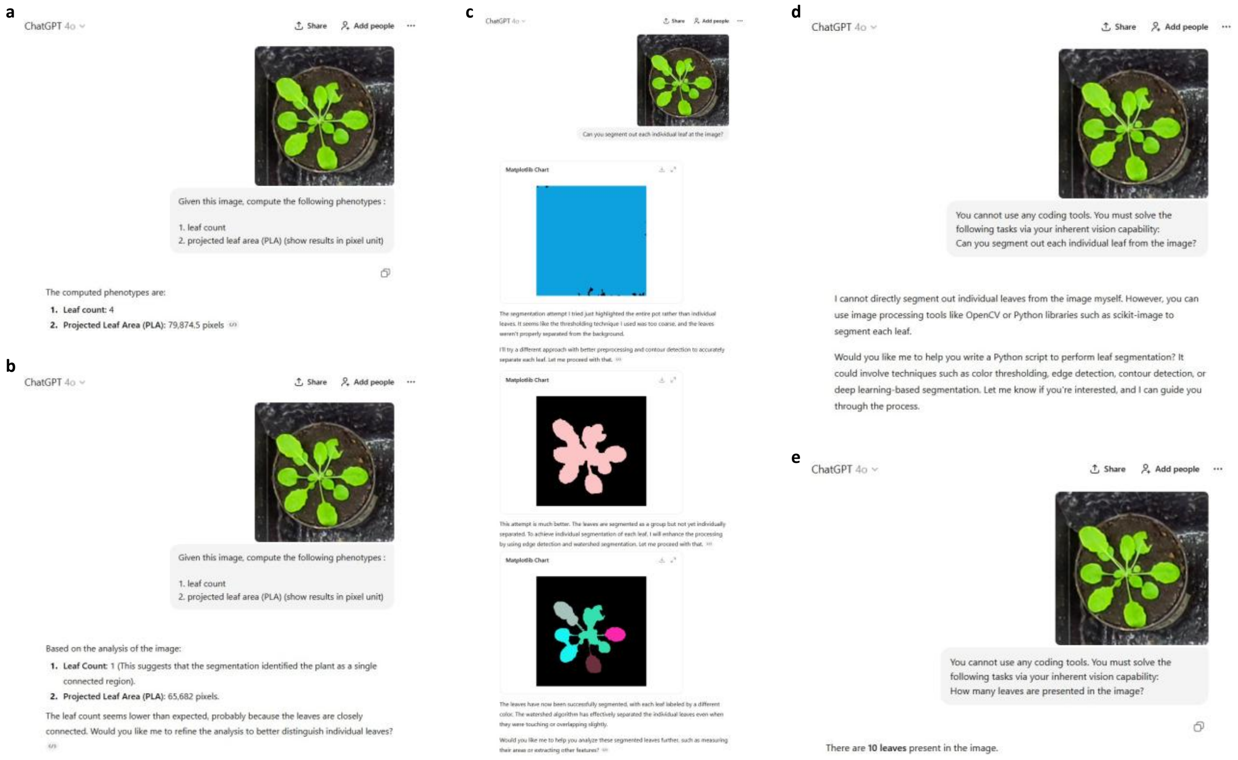

Supplementary Fig. 1. Screen captures of using ChatGPT 4o to extract plant phenotypes and perform computer vision tasks on a top-view image of *A. thaliana*. a, b ChatGPT is prompted to extract projected leaf area and leaf count without additional instructions in two separate attempts. **c** ChatGPT is prompted to perform leaf instance segmentation. **d, e** ChatGPT is prompted to rely solely on its inherent vision capabilities to perform leaf instance segmentation (d) and leaf counting (e).

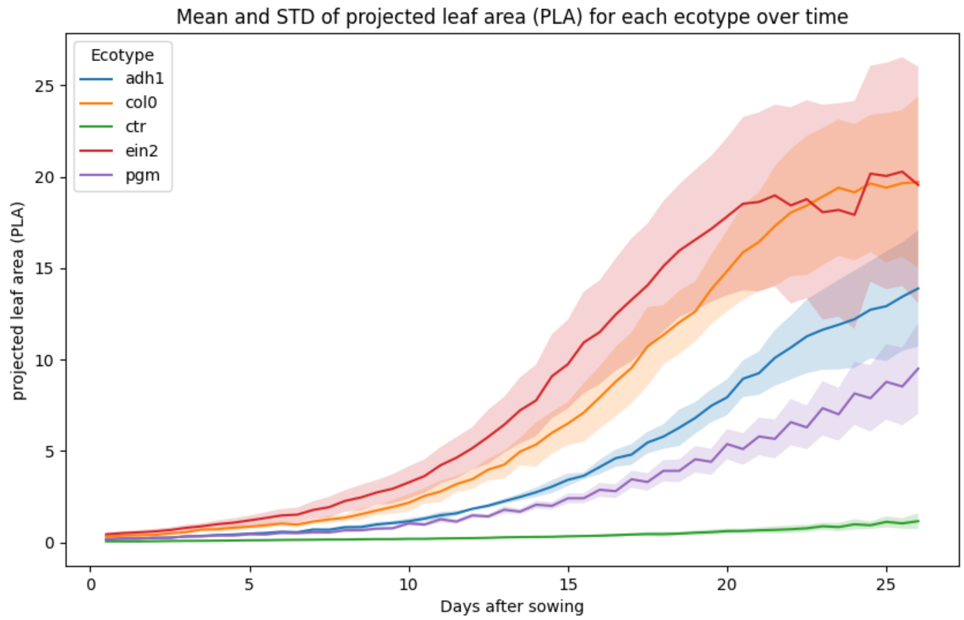

Supplementary Fig. 2. Mean projected leaf area (PLA) over days after sowing. Shaded areas represent the standard deviation (STD) across samples within each ecotype (n = 4 for *adh1*; n = 5 for *col0*, *ctr5*, *ein2*, and *pgm)*. Source data are provided as a Source Data file.

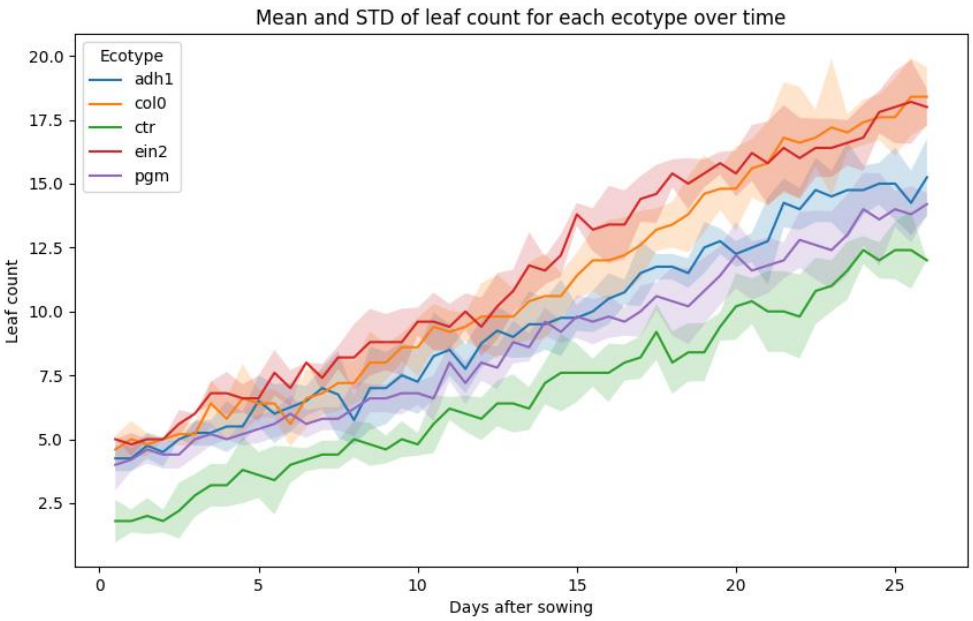

Supplementary Fig. 3. Mean leaf count over days after sowing. Shaded areas represent the standard deviation (STD) across samples within each ecotype (n = 4 for *adh1*; n = 5 for *col0*, *ctr5*, *ein2*, and *pgm)*. Source data are provided as a Source Data file.

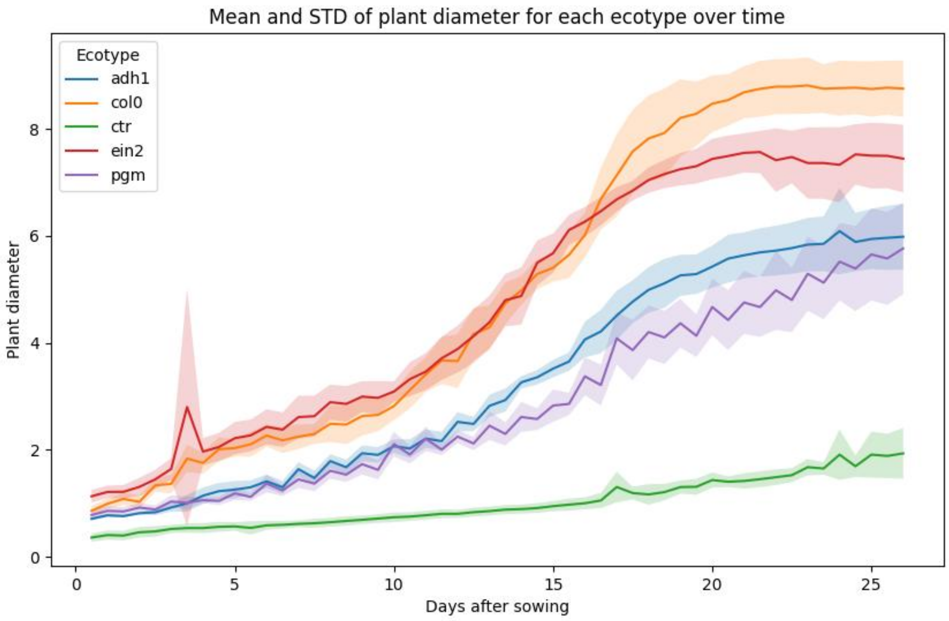

Supplementary Fig. 4. Mean plant diameter over days after sowing. Shaded areas represent the standard deviation (STD) across samples within each ecotype (n = 4 for *adh1*; n = 5 for *col0*, *ctr5*, *ein2*, and *pgm)*. Source data are provided as a Source Data file.

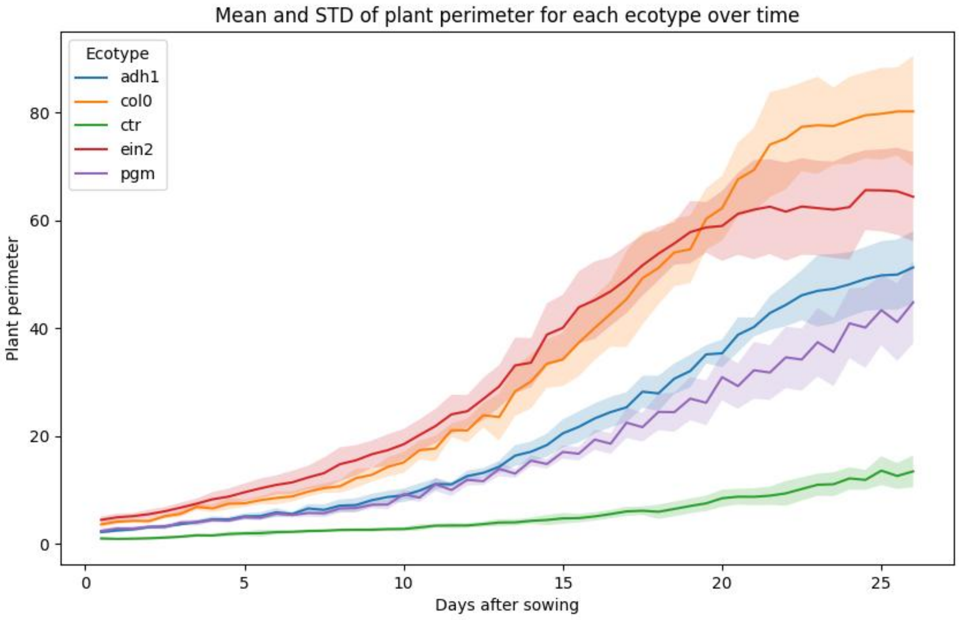

Supplementary Fig. 5. Mean plant perimeter over days after sowing. Shaded areas represent the standard deviation (STD) across samples within each ecotype (n = 4 for *adh1*; n = 5 for *col0*, *ctr5*, *ein2*, and *pgm)*. Source data are provided as a Source Data file.

Supplementary Table 1. Feature comparison between our PhenoAssistant and different LLMs (also including multimodal LLMs, i.e. MLLMs) and LLM-based agent systems for plant science and agriculture. Img. In. denotes whether image inputs are allowed.

**Name**	**Primary Scope**	**Key Ability**	**System Type**	**Core Model**	**Img. In.**
PhenoAssistant (Ours)	Multi-agent system to automate plant phenotyping workflows	Address multi-step phenotyping tasks including phenotype extraction, data visualisation and analysis (e.g. measuring, plotting, and performing statistical tests for leaf-related morphological traits)	Multi-agent	GPT-4o	✓
Agri-bot	Chatbot to provide personalised suggestions on organic farming in Tamil Nadu	RAG-based Q&A on organic farming (e.g. on crop cultivation and pest control)	Single-agent	Cohere command-xlarge-nightly	✗
AgriGenius	Chatbot to address farmer's agricultural queries	RAG-based Q\&A (information retrieved from agricultural websites and documents)	Single-agent	Llama-2-70B	✗
Pllama[1]	LLM pretrained on plant related literature	Plant knowledge Q&A	LLM	Finetuned Llama-2-7B/13B	✗
AgroGPT[2]	Multilingual MLLM for fine-grained agricultural Q&A	Fine-tuned model for agricultural Q&A (e.g. disease and insect identification)	MLLM	LLaVA-7B/Mipha-3B	✓
ShizishanGPT[3]	Q&A system for agricultural knowledge	RAG, searching, graph, agricultural models enhanced agricultural Q&A (e.g. growth or DNA related analysis on maize)	Single-agent	GPT-4	✗
IPM-AgriGPT[4]	LLM for pest and disease management Q&A in Chinese	Domain specific finetuned, and agricultural contextual reasoning chain-of-thought distillation enhanced Q&A (e.g. to recommend proper control measures for specific crop diseases)	LLM	Pre-trained and fine-tuned ChatGLM3-6B	✗
AgroLLM[5]	Chatbot for agriculture and life sciences, agricultural management, agriculture and forestry, and agriculture business	RAG-based knowledge retrieval from agricultural textbooks and research articles to answer conceptual questions on farming and agriculture	Single-agent	Gemini-1.5-Flash, ChatGPT-4o-Mini, and Mistral-7B-Instruct-v0.2	✗
AgEval[6]	Benchmark for MLLMs on plant stress phenotyping	In-context learning based identification, classification, and quantification on plant stress related tasks (e.g. identifying stress types, classifying stress tolerance, and quantifying disease extent)	MLLM	GPT-4o, Claude-3-Haiku/3.5-Sonnet, Gemini-1.5-Flash/Pro, LLaVA v1.6-34B	✓
Orchestrator-Agent Trust[7]	Apple leaf disease diagnosis	Trust-calibrated and RAG enhanced disease classification (e.g. rust and scab) on apple leaf images	Multi-agent	o3-mini, GPT-4o, Qwen-2.5-VL	✓
PlantGPT[8]	Arabidopsis functional-genomics Q&A	A fine-tuned, RAG-enhanced model to answer Arabidopsis gene–phenotype related questions	Single-agent	Finetuned LLama3-8B	✗

Supplementary Table 2. Full evaluation results on tool selection comparing PhenoAssistant's original design (Manager) with the addition of a critic agent. Chain denotes Overall Chain, Exist. denotes Tool Existence, Appro. denotes Tool Appropriateness, Arg. denotes Arguments, Avg denotes the average scores (across aspects or tasks), and Diff. is the difference in the average scores between Manager + Critic and Manager.

**Task**	**Manager**					**Manager + Critic**					**Diff.**
	Chain	Exist.	Appro.	Arg.	Avg	Chain	Exist.	Appro.	Arg.	Avg	
1	4	5	4	4	4.25	4	5	5	4	4.50	0.25
2	4	5	5	3	4.25	4	5	5	4	4.50	0.25
3	4	5	5	4	4.50	4	5	5	4	4.50	0.00
4	4	5	5	5	4.75	4	5	5	4	4.50	-0.25
5	3	5	3	3	3.50	4	5	4	5	4.50	1.00
6	4	5	4	4	4.25	4	5	4	5	4.50	0.25
7	5	5	5	4	4.75	4	5	5	4	4.50	-0.25
8	5	5	5	5	5.00	4	5	5	4	4.50	-0.5
9	5	5	5	5	5.00	5	5	5	5	5.00	0.00
10	4	5	5	4	4.50	4	5	5	4	4.50	0.00
11	4	5	4	4	4.25	4	5	5	4	4.50	0.25
12	2	5	4	5	4.00	4	5	5	3	4.25	0.25
13	5	5	5	5	5.00	5	5	5	5	5.00	0.00
14	5	5	5	5	5.00	5	5	5	5	5.00	0.00
15	5	5	5	4	4.75	5	5	5	5	5.00	0.25
16	3	5	4	3	3.75	4	5	5	4	4.50	0.75
17	5	5	5	5	5.00	5	5	5	5	5.00	0.00
18	5	5	5	5	5.00	5	5	5	5	5.00	0.00
19	5	5	5	5	5.00	5	5	5	5	5.00	0.00
20	4	5	5	4	4.50	4	5	5	4	4.50	0.00
Avg	4.25	5.00	4.65	4.30	4.55	4.35	5.00	4.90	4.40	4.66	0.11

Supplementary references

1. Yang, X., Gao, J., Xue, W. & Alexandersson, E. Pllama: An open-source large language model for plant science. Preprint at https://arxiv.org/abs/2401.01600 (2024).
2. Awais, M., Salem Abdulla Alharthi, A. H., Kumar, A., Cholakkal, H. & Anwer, R. M. AgroGPT : Efficient Agricultural Vision-Language Model with Expert Tuning. in *2025 IEEE/CVF Winter Conference on Applications of Computer Vision (WACV)* 5687–5696 (2025). doi:10.1109/WACV61041.2025.00555.
3. Yang, S. *et al.* ShizishanGPT: An agricultural large language model integrating tools and resources. in *International Conference on Web Information Systems Engineering* 284–298 (Springer, 2024).
4. Zhang, Y. *et al.* IPM-AgriGPT: A Large Language Model for Pest and Disease Management with a G-EA Framework and Agricultural Contextual Reasoning. *Mathematics* **13**, 566 (2025).
5. Samuel, D. J., Skarga-Bandurova, I., Sikolia, D. & Awais, M. AgroLLM: Connecting Farmers and Agricultural Practices through Large Language Models for Enhanced Knowledge Transfer and Practical Application. Preprint at https://arxiv.org/abs/2503.04788 (2025).
6. Arshad, M. A. *et al.* Leveraging Vision Language Models for Specialized Agricultural Tasks. in *2025 IEEE/CVF Winter Conference on Applications of Computer Vision (WACV)* 6320–6329 (2025). doi:10.1109/WACV61041.2025.00616.
7. Roumeliotis, K. I., Sapkota, R., Karkee, M. & Tselikas, N. D. Orchestrator-Agent Trust: A Modular Agentic AI Visual Classification System with Trust-Aware Orchestration and RAG-Based Reasoning. Preprint at https://arxiv.org/abs/2507.10571 (2025).
8. Zhang, R. *et al.* PlantGPT: An Arabidopsis-Based Intelligent Agent that Answers Questions about Plant Functional Genomics. *Advanced Science* **12**, e03926 (2025).
