# Supplementary material for: A conversational multi-agent AI system for automated plant phenotyping
Source: Nat Commun. 2026 Apr 3;17:6391. doi: 10.1038/s41467-026-71090-y (PMC13376813; doi:10.1038/s41467-026-71090-y)
Supplement: Supplementary file 3 — Reporting Summary [file 41467_2026_71090_MOESM3_ESM.pdf]

nature portfolio

Corresponding author(s): Feng Chen, Sotirios A. Tsaftaris, Mario Valerio Giuffrida

Last updated by author(s): Jan 21, 2026

Reporting Summary

Nature Portfolio wishes to improve the reproducibility of the work that we publish. This form provides structure for consistency and transparency in reporting. For further information on Nature Portfolio policies, see our Editorial Policies and the Editorial Policy Checklist.

Statistics

For all statistical analyses, confirm that the following items are present in the figure legend, table legend, main text, or Methods section.

n/a	Confirmed	
☐	☒	The exact sample size (*n*) for each experimental group/condition, given as a discrete number and unit of measurement
☒	☐	A statement on whether measurements were taken from distinct samples or whether the same sample was measured repeatedly
☐	☒	The statistical test(s) used AND whether they are one- or two-sided<br>*Only common tests should be described solely by name; describe more complex techniques in the Methods section.*
☒	☐	A description of all covariates tested
☒	☐	A description of any assumptions or corrections, such as tests of normality and adjustment for multiple comparisons
☒	☐	A full description of the statistical parameters including central tendency (e.g. means) or other basic estimates (e.g. regression coefficient) AND variation (e.g. standard deviation) or associated estimates of uncertainty (e.g. confidence intervals)
☐	☒	For null hypothesis testing, the test statistic (e.g. *F*, *t*, *r*) with confidence intervals, effect sizes, degrees of freedom and *P* value noted<br>*Give P values as exact values whenever suitable.*
☒	☐	For Bayesian analysis, information on the choice of priors and Markov chain Monte Carlo settings
☒	☐	For hierarchical and complex designs, identification of the appropriate level for tests and full reporting of outcomes
☐	☒	Estimates of effect sizes (e.g. Cohen's *d*, Pearson's *r*), indicating how they were calculated

Our web collection on statistics for biologists contains articles on many of the points above.

Software and code

Policy information about availability of computer code

Data collection	https://github.com/vios-s/PhenoAssistant/ (main packages: python 3.11.10, autogen-agentchat 0.2.39, pytorch 2.4.1, transformers 4.45.2, pandasai 2.4.2)
Data analysis	https://github.com/vios-s/PhenoAssistant/ (main packages: python 3.11.10, autogen-agentchat 0.2.39, pytorch 2.4.1, transformers 4.45.2, pandasai 2.4.2)

For manuscripts utilizing custom algorithms or software that are central to the research but not yet described in published literature, software must be made available to editors and reviewers. We strongly encourage code deposition in a community repository (e.g. GitHub). See the Nature Portfolio guidelines for submitting code & software for further information.

Data

Policy information about availability of data

All manuscripts must include a data availability statement. This statement should provide the following information, where applicable:

- Accession codes, unique identifiers, or web links for publicly available datasets
- A description of any restrictions on data availability
- For clinical datasets or third party data, please ensure that the statement adheres to our policy

Data availability: The data that support the findings of Case Study 1 are available from http://phenotiki.com/ but restrictions apply to the availability of these data, which were used under license for the current study, and so are not publicly available. Data are however available from the authors upon reasonable request and with permission of http://phenotiki.com/. The dataset for training the computer vision model used in Case Study 1 is available in CVPPP2017 Leaf Segmentation

Challenge repository, https://codalab.lisn.upsaclay.fr/competitions/8970. The data that support the findings of Case Study 2 are available at Potato Leaf data set repository, https://zenodo.org/records/7938231. The data that support the findings of Case Study 3 are available at CVPPA@ICCV'23: Image Classification of Nutrient Deficiencies in Winter Wheat and Winter Rye repository, https://codalab.lisn.upsaclay.fr/competitions/13833.

Research involving human participants, their data, or biological material

Policy information about studies with human participants or human data. See also policy information about sex, gender (identity/presentation), and sexual orientation and race, ethnicity and racism.

Reporting on sex and gender	does not apply
Reporting on race, ethnicity, or other socially relevant groupings	does not apply
Population characteristics	does not apply
Recruitment	does not apply
Ethics oversight	does not apply

Note that full information on the approval of the study protocol must also be provided in the manuscript.

Field-specific reporting

Please select the one below that is the best fit for your research. If you are not sure, read the appropriate sections before making your selection.

[x] Life sciences [ ] Behavioural & social sciences [ ] Ecological, evolutionary & environmental sciences

For a reference copy of the document with all sections, see nature.com/documents/nr-reporting-summary-flat.pdf

Life sciences study design

All studies must disclose on these points even when the disclosure is negative.

Sample size	Sample sizes for case studies were determined via the data source (see Data section). Sample sizes for evaluation were determined considering model inference time, human evaluation costs, and the need to cover a representative range for system assessment.
Data exclusions	No data were excluded.
Replication	Replication is possible by following the same environment configuration and prompts provided at https://github.com/vios-s/PhenoAssistant/. We also provide the full chat history that determined the content of the manuscript in the same repository. Given that our system relies on large language models and deep learning, which inherently involve some degree of randomness, replication may result in variations.
Randomization	This is not relevant to our study. All data for case studies are from existing publications. All tasks for system evaluation are assessed.
Blinding	This is not relevant to our study. All data for case studies are from existing publications. System evaluation tasks are designed to cover a representative range for system assessment.

Reporting for specific materials, systems and methods

We require information from authors about some types of materials, experimental systems and methods used in many studies. Here, indicate whether each material, system or method listed is relevant to your study. If you are not sure if a list item applies to your research, read the appropriate section before selecting a response.

Materials & experimental systems

n/a	Involved in the study	
[x]	[ ]	Antibodies
[x]	[ ]	Eukaryotic cell lines
[x]	[ ]	Palaeontology and archaeology
[x]	[ ]	Animals and other organisms
[x]	[ ]	Clinical data
[x]	[ ]	Dual use research of concern
[ ]	[x]	Plants

Methods

n/a	Involved in the study	
[x]	[ ]	ChIP-seq
[x]	[ ]	Flow cytometry
[x]	[ ]	MRI-based neuroimaging

Dual use research of concern

Policy information about dual use research of concern

Hazards

Could the accidental, deliberate or reckless misuse of agents or technologies generated in the work, or the application of information presented in the manuscript, pose a threat to:

No	Yes	
☒	☐	Public health
☒	☐	National security
☒	☐	Crops and/or livestock
☒	☐	Ecosystems
☒	☐	Any other significant area

Experiments of concern

Does the work involve any of these experiments of concern:

No	Yes	
☒	☐	Demonstrate how to render a vaccine ineffective
☒	☐	Confer resistance to therapeutically useful antibiotics or antiviral agents
☒	☐	Enhance the virulence of a pathogen or render a nonpathogen virulent
☒	☐	Increase transmissibility of a pathogen
☒	☐	Alter the host range of a pathogen
☒	☐	Enable evasion of diagnostic/detection modalities
☒	☐	Enable the weaponization of a biological agent or toxin
☒	☐	Any other potentially harmful combination of experiments and agents

Plants

Seed stocks	Not applicable. All data for case studies are from existing publications.
Novel plant genotypes	Not applicable. All data for case studies are from existing publications.
Authentication	Not applicable. All data for case studies are from existing publications.
