# Supplementary material for: A conversational multi-agent AI system for automated plant phenotyping
Source: Nat Commun. 2026 Apr 3;17:6391. doi: 10.1038/s41467-026-71090-y (PMC13376813; doi:10.1038/s41467-026-71090-y)
Supplement: Supplementary file 2 — Peer Review File [file 41467_2026_71090_MOESM2_ESM.pdf]

nature portfolio

Peer Review File

A Conversational Multi-Agent AI System for Automated Plant Phenotyping

Corresponding Author: Dr Mario Giuffrida

This file contains all reviewer reports in order by version, followed by all author rebuttals in order by version.

Version 0:

Reviewer comments:

Reviewer #1

(Remarks to the Author)

This paper presents PhenoAssistant, an innovative AI-driven platform that simplifies plant phenotyping through natural language interfaces. By integrating a large language model (LLM) with a curated toolkit for phenotype extraction, visualization, and model training, the system reduces technical barriers and aim to enhance accessibility for researchers with limited computational expertise. The tool is demonstrated by three case studies and three aspects were evaluated as of its performance. These, both test cases and evaluation tasks, are all reasonable and represents an array of different needs in plant phenotyping. The main value of this paper is the first case to demonstrate AI agent applications for plant phenotyping (the application value). PhenoAssistant fills a gap which is likely needed, "democratising", the use of plant phenotyping. Usage will tell to what extent these types of tool will accelerate the advancement, incorporation and use of plant phenotyping in the plant sciences.

Major comment:

As this is a collection of tools into a kit, there are no clear contribution on new methods or methods as it is mainly based on using GPT-4o, and as such difficult to benchmark against other tools even if an internal evaluation is done on performance. Can the generalizaion ability of the AI agent be ensured by further tests?

Minor comments:
It is not stated who performed the case studies. Probably by the authors who have great expertise in plant phenotyping methods and thinking, and thus could be criticised not to be the target group the PhenoAssistant tool is aimed at.

It could be more clearly stated in the introduction that this tool and other AI agents also bridges different expertise.

To more clearly highlight that that ChatGPT cannot correctly extract phenotypes from images (Supplementary Section A.1), this should be lifted in the text.

How is the tool maintained and envisaged to be developed?

Indicate in the Appendix which specific tasks as part of the evaluation failed.

The methods are even if clearly aimed at non-LLM experts quite generically presented in the methods section. A refinement could be considered.

(Remarks on code availability)

Reviewer #2

(Remarks to the Author)
This is a good paper, and timely needed content. However I have following concerns as a MAJOR concerns before I read

the revised version for further publication:

1) While different agents (e.g., Code Writer, RAG Agent, Data Visualizer) handle specific subtasks, they follow fixed responsibilities. There is no dynamic role negotiation or emergent division of laborhallmarks of Agentic AI.

2) Although multiple agents are involved, they operate under a central LLM "manager" with no decentralized decision-making or peer-to-peer coordination, which constrains scalability and emergent intelligence.

3) The paper lacks a proper literature in introduction to differentiate what AI Agents are and how are AI Agents different than Agentic AI.

4) There is no mechanism for the agents to simulate tasks, explore solution spaces, or verify strategies before execution which is a key requirement for high-assurance applications and agentic behavior.

5) Introduce agent-based simulation tools where agents can virtually test phenotyping hypotheses (e.g., nutrient deficiency responses(this is just an example, you can consider relevant example to your dataset but the example should be applied) before deploying real-world models

6) Try to integrate causal modeling agents that reason about cause-effect relationships in plant physiology and validate hypotheses using both statistical and symbolic methods.

7) Write more discussion on Agentic AI how will it be different than a normal AI Agent, present some recent findings and literatures how they define this both terms and also write the discussion as Scalable Infrastructure and Governance in the future discussion part.

8) You could potentially think about shifting from a central-manager paradigm to decentralized agent collaboration with local autonomy, communication protocols, and a shared task environment—more aligned with Agentic AI frameworks such as ChatDev or CrewAI or more.

(Remarks on code availability)
I will review full code during the revision as I have advised some experimentation revisions too which would need to update the code as well.

Reviewer #3

(Remarks to the Author)
Overall, this is an interesting and timely work that leverages the new wave of Agentic AI pipelines for automate plant phenotyping tasks. However, the main concerns are: (1) evaluation does not seem comprehensive, and (2) unclear how it may scale to add new tools and models to the toolkit. Hence, the claim seems a bit overblown.

Below are some detailed comments and suggestions.
• In Figure 4, i.e., case study 3, what framework is being used to make the models? For example, how is the appropriate model size selected?
• L218: It seems it is only predicting the type of model to select (e.g., instance segmentation, image classification, etc.), but which exact model is selected from the model zoo is not being evaluated.
• The granularity of prediction in invasion model selection and the number of tool selections (currently 10) and data analysis tasks (currently 10 as well) should be evaluated.
• L254: The accuracy of reproduced pipelines should also be given.
• L304: It is rightly noted that it is practically impossible to pre-integrate every required vision model. To solve this problem, a mechanism for dynamically fetching onion models could help. The publicly available open-sourced models with the 'agriculture' tag, for example, could be fetched from Kaggle. https://www.kaggle.com/models?query=agriculture&owner-type=user
• L324: Where is the use of LoRA fine-tuning demonstrated in the presented use cases?
• L580 - L635: The pipeline is supposed to predict one of these labels: 'instance segmentation, image classification, and image regression,' but the inputs themselves give it away. For example, the inputs were: Flower color classification, Leaf disease segmentation, Fruit ripening stage classification. So, even though it is reported that this evaluation is pretty surface level, the reported performance of 98% does not mean much.
• How is the model going to know if the image being asked for is trained for it? VLMs can take general input, but these specialized trained models cannot, so how are they fed? For example, if any image from another dataset is captured which does not correspond to training data, then it is a known limitation with traditional models.
• Make a clear distinction between a module and an agent. For example, why is statistical test listed as an agent?
• Model Context Protocol (MCP) is a standardized way to connect to external data sources and tools through a unified interface. Instead of PhenoAssistant creating its own custom toolkit integration system, they could have used MCP to connect their LLM to the vision models, statistical tools, etc.
• Chat logs and evaluation results are provided in a public repository, but the software/tool itself is not available. Also, it would help to publicly document the method to add new agents in the toolkit, after making it publicly accessible.
• A matrix table containing the feature comparison of PhenoAssistant with other existing tools would

be a great addition. Some tools available online in this space are: https://github.com/Taranshah9/MumbaiHacks2024_LlamaMia https://github.com/nalinrajendran/eco-agri-bot https://github.com/jayeshbhandarkar/AgriGenius

(Remarks on code availability)

Reviewer #4

(Remarks to the Author)
I co-reviewed this manuscript with one of the reviewers who provided the listed reports. This is part of the Nature Communications initiative to facilitate training in peer review and to provide appropriate recognition for Early Career Researchers who co-review manuscripts.

(Remarks on code availability)
I co-reviewed this manuscript with one of the reviewers who provided the listed reports. This is part of the Nature Communications initiative to facilitate training in peer review and to provide appropriate recognition for Early Career Researchers who co-review manuscripts.

Version 1:

Reviewer comments:

Reviewer #1

(Remarks to the Author)
I am pleased with the extensive response and also the changes made to the manuscript based on my comments. I have no further comments

(Remarks on code availability)

Reviewer #2

(Remarks to the Author)
Great Revision. However, there are still spaces to improve the paper with minor and important changes

In the abstract, a proper GitHub repository link could be added as the last sentence.

Figure 2, 3 and 4 caption could be more detailed for better read(basic to advanced reader audience should be focused for captioning such figures)

Figure 5, the leaf count, plant diameter and plant perimeter graphs are hard to see and understand even with glasses. Please enlarge all those graphs to PLA size graph at least for better visibility

(Remarks on code availability)
You could add some images of manuscript in the github page too, maintain page like a blog kind of information too with images that shows a proper instruction about the paper....

Reviewer #3

(Remarks to the Author)
Here are my feedback on the authors' responses to my questions.

• In Figure 4, i.e., case study 3, what framework is being used to make the models? For example, how is the appropriate model size selected?
Response Assessment:
– Satisfied/Unsatisfied: Satisfied
– Additional Comments: I agree the model size and optimizations become secondary in this context. The authors say "we do integrate both LoRA [39] and full-parameter finetuning options to allow users adapt to their hardware (e.g. GPUs), dataset size, and requirements for model performance." However, there is a caveat that it takes more effort on the logistical side of setting up everything on the computer, which could vary from one system to another, to use the full capability of the hardware.

• L218: It seems it is only predicting the type of model to select (e.g., instance segmentation, image classification, etc.), but which exact model is selected from the model zoo is not being evaluated.

Response Assessment:
– Satisfied/Unsatisfied: Unsatisfied
– Additional Comments: While I acknowledge the authors' explanation that they evaluate model type prediction rather than exact model selection, this evaluation task is too simplistic to properly assess the framework's capabilities. The high accuracy numbers reported don't provide meaningful confidence in the system because the task of categorizing into broad model types (instance segmentation, image classification, etc.) is relatively easy. A more challenging evaluation would better demonstrate the system's real-world utility.

• The granularity of prediction in invasion model selection and the number of tool selections (currently 10) and data analysis tasks (currently 10 as well) should be evaluated.
Response Assessment:
– Satisfied/Unsatisfied: Satisfied
– Additional Comments: I acknowledge that the authors added meaningful numbers and evaluation details for the current scope of their toolkit. However, this does not fully address the scalability issue since the process remains manual. For the scope of this paper, the evaluation is adequate, but this manual limitation should be noted as a caveat for future scalability.

• L254: The accuracy of reproduced pipelines should also be given.
Response Assessment:
– Satisfied/Unsatisfied: Indifferent
– Additional Comments: Case study 1 provides anecdotal evidence that sparks confidence, but reproduction is limited to one case only.

• L304: It is rightly noted that it is practically impossible to pre-integrate every required vision model. To solve this problem, a mechanism for dynamically fetching onion models could help. The publicly available open-sourced models with the 'agriculture' tag, for example, could be fetched from Kaggle. https://www.kaggle.com/models?query=agriculture&owner-type=user
Response Assessment:
– Satisfied/Unsatisfied: Satisfied
– Additional Comments: The authors rightly noted the limitations of fetching online models and thus pre-fetching is indeed necessary in this context to add reliability.

• L324: Where is the use of LoRA fine-tuning demonstrated in the presented use cases?
Response Assessment:
– Satisfied/Unsatisfied: Satisfied
– Additional Comments: It has now been added in revision.

• L580 - L635: The pipeline is supposed to predict one of these labels: 'instance segmentation, image classification, and image regression,' but the inputs themselves give it away. For example, the inputs were: Flower color classification, Leaf disease segmentation, Fruit ripening stage classification. So, even though it is reported that this evaluation is pretty surface level, the reported performance of 98% does not mean much.
Response Assessment:
– Satisfied/Unsatisfied: Satisfied
– Additional Comments: The list of evaluation tasks has been diversified. However, the question of using this as a metric to validate the framework is still questionable (as shared above). But I am satisfied with the concern of having revealing labels in the input.

• How is the model going to know if the image being asked for is trained for it? VLMs can take general input, but these specialized trained models cannot, so how are they fed? For example, if any image from another dataset is captured which does not correspond to training data, then it is a known limitation with traditional models.
Response Assessment:
– Satisfied/Unsatisfied: Indifferent
– Additional Comments: This is rightly noted as a limitation (out of distribution). However, the expectation that experts will be able to identify issues from intermediary outputs is not reasonable because images could be taken from different cameras and human eye/expert cursory evaluation might not catch such cases. This could be a low hanging fruit for further improvement. If the system could detect out of distribution cases, that would add hugely to the reliability of the system.

• Draw a line between calling it a module and an agent. For example, why is statistical test listed as an agent
Response Assessment:
– Satisfied/Unsatisfied: Satisfied
– Additional Comments: Thanks for clarifying in text.

• Model Context Protocol (MCP) is a standardized way to connect to external data sources and tools through a unified interface. Instead of PhenoAssistant creating its own custom toolkit integration system, they could have used MCP to connect their LLM to the vision models, statistical tools, etc.
Response Assessment:
– Satisfied/Unsatisfied: Satisfied

– Additional Comments: The authors acknowledge MCP as a valid alternative but note their custom integration was developed before MCP became widely adopted. While MCP would indeed reduce redundant integration code, the current system’s modular design allows for migration to MCP in future versions.
The code is now public on GitHub along with v1 and v2 releases. Instructions to run the tool and add new agents are included. Chat logs and evaluation results are also available.

• A matrix table containing the feature comparison of PhenoAssistant with other existing tools would be a great addition. Some tools available online in this space are: https://github.com/Taranshah9/MumbaiHacks2024_LlamaMia https://github.com/nalinrajendran/eco-agri-bot https://github.com/jayeshbhandarkar/AgriGenius
Response Assessment:
– Satisfied/Unsatisfied: Satisfied
– Additional Comments: The table includes both benchmarks and tools, but it still gives a good overview. And I meant overview in terms of features, which could be more granular, such as specifying the types of phenotyping tasks or plant trait assessments supported.

(Remarks on code availability)

Reviewer #4

(Remarks to the Author)
I co-reviewed this manuscript with one of the reviewers who provided the listed reports. This is part of the Nature Communications initiative to facilitate training in peer review and to provide appropriate recognition for Early Career Researchers who co-review manuscripts.

(Remarks on code availability)
I co-reviewed this manuscript with one of the reviewers who provided the listed reports. This is part of the Nature Communications initiative to facilitate training in peer review and to provide appropriate recognition for Early Career Researchers who co-review manuscripts.

Version 2:

Reviewer comments:

Reviewer #3

(Remarks to the Author)
I am happy with the careful responses from the authors, no further comments.

(Remarks on code availability)

Open Access This Peer Review File is licensed under a Creative Commons Attribution 4.0 International License, which permits use, sharing, adaptation, distribution and reproduction in any medium or format, as long as you give appropriate credit to the original author(s) and the source, provide a link to the Creative Commons license, and indicate if changes were made.
In cases where reviewers are anonymous, credit should be given to 'Anonymous Referee' and the source.
The images or other third party material in this Peer Review File are included in the article's Creative Commons license, unless indicated otherwise in a credit line to the material. If material is not included in the article's Creative Commons license and your intended use is not permitted by statutory regulation or exceeds the permitted use, you will need to obtain permission directly from the copyright holder.
To view a copy of this license, visit https://creativecommons.org/licenses/by/4.0/

Responses to Reviewers' Comments

To All Reviewers:

We greatly appreciate your recognition of the value of our work and sincerely thank you for the time and effort dedicated to reading and reviewing our manuscript. Taking the input from the reviewers, we have now submitted a revised manuscript and supplementary information. Furthermore, we provide a point-by-point response to all the concerns raised by the reviewers below.

Before reading our responses, we kindly ask you to read the following notes:

Note 1. How changes are indicated in the revised manuscript:

Wherever a response required modifications to the manuscript, the corresponding changes have been **highlighted in Orange and labelled with R*m.n***, where *m* refers to the index of reviewer, and *n* represents the index of comment. For example, R1.2 refers to Reviewer 1's Comment 2.

We also revised other parts of the manuscript to better fit the context while addressing reviewers' comments, correct typographical errors, and ensure consistent word usage. These changes **are highlighted in Orange but not necessarily labelled with R*m.n***.

Note 2. Reorganisation of sections:

To comply with the formatting requirements of *Nature Communications*, we reorganised these sections:

(i) We created a new Section 2 Results by consolidating the original Section 2 Design of PhenoAssistant (now Section 2.1), Section 3 Case Studies (now Section 2.2), and Section 4 Evaluation of PhenoAssistant (now Section 2.3).

(ii) The layout of the new Section 2.3 Evaluation of PhenoAssistant has been revised for better clarity and comprehensiveness. Previously, we presented only a brief description of each evaluation and then discussed the results collectively at the end. In the new version, we have expanded the descriptions, discussed the results of each evaluation in detail separately, and provided a summary at the end.

(iii) We created a new Section 3 Discussion by merging the original Section 5 (Conclusion) with additional discussion on PhenoAssistant's limitations and potential future directions.

(iv) We moved the original Appendix A Supplementary Materials into a separate file titled Supplementary Information. The section labels in this new file have been updated accordingly (e.g. previously: A.2 Chat Log for Case Study 1 Task 1, now: Supplementary Note 2 Chat Log for Case Study 1 Task 1), and the corresponding cross-references in the main text have been updated.

Reviewer #1 (Remarks to the Author):

R1.0: This paper presents PhenoAssistant, an innovative AI-driven platform that simplifies plant phenotyping through natural language interfaces. By integrating a large language model (LLM) with a curated toolkit for phenotype extraction, visualization, and model training, the system reduces technical barriers and aim to enhance accessibility for researchers with limited computational expertise. The tool is demonstrated by three case studies and three aspects were evaluated as of its performance. These, both test cases and evaluation tasks, are all reasonable and represents an array of different needs in plant phenotyping. The main value of this paper is the first case to demonstrate AI agent applications for plant phenotyping (the application value). PhenoAssistant fills a gap which is likely needed, "democratising", the use of plant phenotyping. Usage will tell to what extent these types of tool will accelerate the advancement, incorporation and use of plant phenotyping in the plant sciences.

Response: We thank you for the positive and encouraging assessment of our work. We greatly appreciate your recognition of the novelty of PhenoAssistant, the reasonable design of the case studies and evaluations, its application value, and its potential to democratise plant phenotyping. In the following, we address each of your comments point by point.

Major comment:

R1.1: As this is a collection of tools into a kit, there are no clear contribution on new methods or methods as it is mainly based on using GPT-4o, and as such difficult to benchmark against other tools even if an internal evaluation is done on performance. Can the generalizaion ability of the AI agent be ensured by further tests?

Response: Thank you for your comment.

Regarding contributions:

First, we would like to clarify that our work is not merely a collection of tools, or a simple application of an LLM (i.e. GPT-4o) on plant phenotyping. Our work is a pioneering study to demonstrate that a multi-agent AI system can lower technical barriers and streamline plant phenotyping workflows. Achieving this required substantial methodological design and implementation beyond simply integrating GPT-4o with existing tools.

To be clearer on what is an LLM, and how an LLM can be augmented to an LLM agent, and then to a multi-agent AI system, we have edited **Lines 482 - 502 at Section 4.1 Preliminaries: LLMs and Agents**.

As shown in Figure 1, PhenoAssistant was designed around a centralised manager agent that coordinates all task execution, combined with a human-in-the-loop mechanism that allows users to approve or correct intermediate steps. This design was introduced not by accident, but to meet two critical requirements: (i) the safety and reliability demanded by plant phenotyping, and (ii) the current limitations of LLMs, which often require oversight and correction.

PhenoAssistant's toolkit is not a simple aggregation of random tools but was designed to support the needs of plant phenotyping, for example, to extract phenotypes from images (computer vision models), to analyse phenotypes (the Table Analyser agent), to reproduce pipelines (the Pipeline Reproducer agent), and to adapt to new scenarios (automatic model training). Once developed, the tools were not automatically “plugged into” PhenoAssistant. Instead, each tool required a carefully written description and input/output specification so that the manager agent can chain and invoke them properly to complete a task. The entire

system went through multiple rounds of design, testing, and refinement to ensure it worked as intended.

Regarding generalisation ability:

Benchmarking a domain-specific AI agent system like PhenoAssistant is indeed challenging. There are not yet widely agreed task sets for such assessments because AI agent systems are emerging, and different systems are designed for different purposes, which makes direct comparisons difficult.

Given that the primary scope of PhenoAssistant is to generate and execute plant phenotyping workflows, we define generalisation ability as the capacity of the system to handle a broader range of phenotyping-related tasks beyond the demonstrated case studies. This heavily relies on both the capability and the coverage of the toolkit. Accordingly, we consider generalisation ability to be captured by two aspects:

Aspect 1: The potential of leveraging the current toolkit to solve new plant phenotyping tasks. Our original evaluations on *tool selection* and *data analysis* already demonstrate this potential. Tool selection tests whether PhenoAssistant can correctly chain existing tools to address a wider range of phenotyping tasks, while data analysis assesses its ability to carry out more analytical tasks.

In this revision, we have strengthened these evaluations further. Specifically, the number of tool selection tasks has been doubled from 10 to 20 to cover more plant species and phenotyping scenarios. In addition, we replaced the original manual assessment method for tool selection tasks with an LLM-based evaluator, because the previous method was less comprehensive (providing only binary labels: correct or wrong) and impractical for potential users without detailed knowledge of PhenoAssistant's toolkit. This LLM-based evaluator is a prompted GPT-4o, scoring PhenoAssisant's responses along four dimensions: Overall Chain Correctness, Tool Existence Correctness, Tool Appropriateness Correctness, and Argument Correctness. It not only provides more comprehensive results, but more importantly, it allows potential users to easily assess PhenoAssistant on new phenotyping tasks using a consistent evaluation method.

We have revised **Lines 283 - 313** and added **Table 1 (the "Manager" row)** to include the new tool selection evaluation process and results. The prompt to start the evaluation has been revised at **Supplementary Note 4.1 (Lines 114 – 122)**, and the newly added tasks are presented at **Supplementary Note 4.1 (Task 11 – 20)**. The system prompt of the LLM-based evaluator is added to **Supplementary Note 5 Tool Evaluator**.

In terms of the evaluation on data analysis, we have also doubled the number of tasks from 10 to 20 to include more categories (analysing plots, converting file formats, computing and analysing new metrics).

We have revised **Lines 341 - 351** to discuss the data analysis evaluation on this extended task set. The newly added tasks are presented at **Supplementary Note 4.3 (Task 11 – 20)**. Please note that Task 17 and 18 fall into a previously existed but less represented category.

Aspect 2: Extendibility of the toolkit. PhenoAssistant is equipped with automatic model training functions to expand its model zoo for new phenotype extraction needs (as demonstrated at Case Study 3). The evaluation on *vision model selection* further tests whether the system can suggest the correct vision model to train for a wider range of plant tasks. To make the evaluation resemble human-agent conversation more closely, we have substantially refined the prompt used in this evaluation to increase linguistic diversity by

expressing the tasks in varied forms, including questions and statements, rather than presenting short phrases only. We have also improved the prompt to prevent information leakage about the target vision models and clarify some previously ambiguous tasks. With these updates, we discuss the new results in **Lines 327 - 340**. The updated prompt is provided at **Supplementary Note 4.2 Lines 279 – 346.**

We believe that these largely updated evaluations can further test PhenoAssistant's generalisation ability, as well as providing potential users a convenient way to assess PhenoAssistant on additional scenarios.

During this revision, we have also included functionalities to support potential users to add additional tools and agents to PhenoAssistant, which are documented in our code repository.

Minor comments:

R1.2: It is not stated who performed the case studies. Probably by the authors who have great expertise in plant phenotyping methods and thinking, and thus could be criticised not to be the target group the PhenoAssistant tool is aimed at.

Response: Thank you for your comment.

Our target group is plant phenotyping researchers and practitioners who attempt to reduce the burden of complex computing and coding. To clarify this point, we have revised multiple places in the manuscript: **Lines 58, 107 – 110, 358 – 360, 477 – 478.**

Our co-authors come from diverse scientific and technical backgrounds, including computer scientists and plant phenotyping researchers with varying levels of computational expertise. The case studies were collaboratively designed and reviewed by several co-authors.

In particular, the prompts (i.e. task descriptions) used to drive the case studies were formulated to reflect the perspective and language of our target group. For example, the prompts use more domain-specific terms such as extracting “leaf count” and “projected leaf area”, rather than computer vision terminology such as “instance segmentation”. The prompts for all case studies are reviewed by several co-authors who have years of experience in plant phenotyping. An example of these prompts is provided in **Supplementary Note 2.** All the other prompts for case studies can be found in our code repository.

We also presented the early design of our case studies at the UK Phenomics Town Hall and Conference 2024 (https://phenomuk.org/conference/), where many attendees represented PhenoAssistant’s intended user community. The feedback was highly positive, with participants enthusiastic about the PhenoAssistant’s usefulness and potential impact.

R1.3: It could be more clearly stated in the introduction that this tool and other AI agents also bridges different expertise.

Response: Thank you for your suggestion.

We have followed your advice and revised accordingly in **Section 1 Introduction: Lines 66, 97 – 103, 123 – 125**.

R1.4: To more clearly highlight that that ChatGPT cannot correctly extract phenotypes from images (Supplementary Section A.1), this should be lifted in the text.

Response: Thank you for your suggestion.

We have followed your recommendation and moved Supplementary Section A.1 from the original manuscript into the main text, placing it before the introduction of PhenoAssistant's vision model zoo. The images from the former Supplementary Section A.1 are now presented as Fig. 2–4.

We have revised several parts in the main text to better integrate this material into the main text's context. Please see **Lines 151 – 175**.

R1.5: How is the tool maintained and envisaged to be developed?

Response: Thank you for your comment.

The source code of PhenoAssistant, along with the case studies and evaluations presented in our paper, is available at https://github.com/fengchen025/PhenoAssistant/. The repository includes documentation on how to install and use PhenoAssistant, as well as instructions for adding new tools and agents, with the goal of enabling plant phenotyping researchers to extend PhenoAssistant for their own research needs.

We welcome contributions from researchers in AI, plant phenotyping, and the wider plant science community to diversify PhenoAssistant's capabilities and to ensure its continued development.

Beyond open sourcing the system, we are actively engaging with the plant phenotyping community (e.g. PhenomUK) to better understand their evolving needs and priorities, thereby guiding the future development of PhenoAssistant. This includes expanding the toolkit to address identified requirements and keeping pace with advances in LLMs and agents such as upgrading the underlying LLM to a more capable.

R1.6: Indicate in the Appendix which specific tasks as part of the evaluation failed.

Response: Thank you for your suggestion.

As noted in our response for R1.1, we have introduced a new evaluation method for tool selection tasks and expanded or refined the tasks across all three evaluations.

For the tool selection evaluation, results are now reported as scores rather than binary correct/incorrect labels. The scores for each task are provided in **Supplementary Table 2 under the "Manager" column**.

There are no failed cases for the vision model selection evaluation; therefore, we included PhenoAssistant's responses in **Supplementary Note 4.2: Lines 347 – 403**.

For the data analysis evaluation, Tasks 11, 12, and 14 failed. We summarised PhenoAssistant's responses and provided the corresponding correct answers in **Supplementary Note 4.3 Task 11, 12, 14**.

R1.7: The methods are even if clearly aimed at non-LLM experts quite generically presented in the methods section. A refinement could be considered.

Response: Thank you for your suggestion.

We have revised the Methods section to be more technical and specific to PhenoAssistant, while still aiming non-LLM background readers. These changes are at **Lines 482 – 502, 507 – 521, 577 - 601**.

Reviewer #2 (Remarks to the Author):

R2.0: This is a good paper, and timely needed content. However I have following concerns as a MAJOR concerns before I read the revised version for further publication:

Response: We thank you for recognising the value and timeliness our work. We appreciate your constructive feedback and address all your concerns in the following responses. Please note that we reorder and group some of your comments when we address them, for better coherence.

R2.3: The paper lacks a proper literature in introduction to differentiate what AI Agents are and how are AI Agents different than Agentic AI.

Response: Thank you for your comment. We would like to address this point first because it is important to position our study properly under the evolving taxonomies of LLMs and agents.

We have followed your recommendation and added a paragraph introducing and comparing AI agents and agentic AI. We also introduced multi-agent AI system, which aligns more closely with our work: it often handles more complex, multi-step workflows than AI agent but exhibit less autonomy and emergent intelligence than agentic AI. This paragraph has been added at **Lines 77 - 94**, which mainly drew upon these new references (numbered as in the revised manuscript):

[10] Sado, F., Loo, C. K., Liew, W. S., Kerzel, M. & Wermter, S. Explainable goal-driven agents and robots-a comprehensive review. ACM Computing Surveys 55, 1–41 (2023).

[11] Acharya, D. B., Kuppan, K. & Divya, B. Agentic ai: Autonomous intelligence for complex goals–a comprehensive survey. IEEE Access (2025).

[12] Sapkota, R., Roumeliotis, K. I. & Karkee, M. Ai agents vs. agentic ai: A conceptual taxonomy, applications and challenges. Information Fusion 126, 103599 (2026).

[13] Hughes, L. et al. Ai agents and agentic systems: A multi-expert analysis. Journal of Computer Information Systems 1–29 (2025)

[14] Borghoff, U. M., Bottoni, P. & Pareschi, R. Human-artificial interaction in the age of agentic ai: a system-theoretical approach. Frontiers in Human Dynamics 7, 1579166 (2025)

We also added some new references that provide more examples of LLMs and agents on healthcare, plant science, and agriculture, revising **Lines 97, 104 – 105**. These are the newly added references (numbered as in the revised manuscript):

[21] Tu, T. et al. Towards conversational diagnostic artificial intelligence. Nature 1–9 (2025)

[22] Singhal, K. et al. Toward expert-level medical question answering with large language models. Nature Medicine 31, 943–950 (2025).

[23] Gottweis, J. et al. Towards an ai co-scientist. arXiv preprint arXiv:2502.18864 (2025).

[25] Awais, M., Alharthi, A. H. S. A., Kumar, A., Cholakkal, H. & Anwer, R. M. Agrogpt: Efficient agricultural vision-language model with expert tuning. 2025 IEEE/CVF Winter Conference on Applications of Computer Vision (WACV), 5687–5696 (IEEE, 2025).

[26] Yang, S. et al. Shizishangpt: An agricultural large language model integrating tools and resources. International Conference on Web Information SystemsEngineering, 284–298 (Springer, 2024).

[27] Zhang, Y. et al. Ipm-agrigpt: A large language model for pest and disease management with a g-ea framework and agricultural contextual reasoning. Mathematics (2227-7390) 13 (2025).

[28] Samuel, D. J., Skarga-Bandurova, I., Sikolia, D. & Awais, M. Agrollm: Connecting farmers and agricultural practices through large language models for enhanced knowledge transfer and practical application. arXiv preprint arXiv:2503.04788 (2025).

[29] Arshad, M. A. et al. Ageval: A benchmark for zero-shot and few-shot plant stress phenotyping with multimodal llms. arXiv preprint arXiv:2407.19617 (2024).

[30] Zhao, X. et al. Implementation of large language models and agricultural knowledge graphs for efficient plant disease detection. Agriculture 14, 1359 (2024).

[31] Roumeliotis, K. I., Sapkota, R., Karkee, M. & Tselikas, N. D. Orchestrator-agent trust: A modular agentic ai visual classification system with trust-awareorchestration and rag-based reasoning. arXiv preprint arXiv:2507.10571 (2025).

[32] Sapkota, R., Roumeliotis, K. I. & Karkee, M. Uavs meet agentic ai: A multidomain survey of autonomous aerial intelligence and agentic uavs. arXiv arXiv:2506.08045 (2025).

[35] Zhang, R. et al. Plantgpt: An arabidopsis-based intelligent agent that answers questions about plant functional genomics. Advanced Science e03926 (2025)

R2.1: While different agents (e.g., Code Writer, RAG Agent, Data Visualizer) handle specific subtasks, they follow fixed responsibilities. There is no dynamic role negotiation or emergent division of laborhallmarks of Agentic AI.

R2.2: Although multiple agents are involved, they operate under a central LLM "manager" with no decentralized decision-making or peer-to-peer coordination, which constrains scalability and emergent intelligence.

R2.8: You could potentially think about shifting from a central-manager paradigm to decentralized agent collaboration with local autonomy, communication protocols, and a shared task environment—more aligned with Agentic AI frameworks such as ChatDev or CrewAI or more.

Response: Thank you for your comment. We would like to address R2.1, R2.2, and R2.7 together as they are closely related.

The goal of PhenoAssistant is to generate and execute workflows for phenotype extraction and analysis. To achieve this, we employ a centralised manager agent that plans and orchestrates the workflow and a small set of specialist agents. These agents have well-scoped and fixed responsibilities to address the major needs of phenotype analysis (e.g. knowledge retrieval, data visualising, and plotting). We choose this centralised and fixed-role design because it already satisfies the goal of PhenoAssistant, while also reducing

coordination complexities, lowering the risk of errors from dynamic role changes, making the overall process more predictable, reproducible, and controllable, which are important in plant phenotyping related research. In addition, as we mentioned in the response to your comment R2.3, we have specified that PhenoAssistant is a multi-agent AI system with less autonomy and scalable intelligence (but enough for our current objectives) than what are typically associated with the evolving definition of agentic AI.

We clarified the reason of using a centralised design with fixed-role agents at the beginning of **Section 2.1 Design of PhenoAssistant: Lines 130 – 131, 134 - 142**.

Decentralised orchestration allows peer-to-peer communications, while dynamic role negotiation (or emergent division of labour) enables self-assign or discover roles through interaction between agents. They can be useful when objectives are creative, open-ended, rapidly changing, or where the appropriate role assignments are unknown beforehand. However, such mechanisms were not prioritised after discussion with our user groups and were **not deemed in scope**. These mechanisms may also introduce costs and risks that we aimed to avoid, for example, increased inference time due to added workflow coordination and role negotiation overhead, system instability or inefficiency when roles are poorly defined, and potential safety concerns.

Hence, while decentralised agent collaboration and dynamic role negotiation are promising and useful for certain agent systems, it is not necessary for our current objectives and may reduce the reliability of our system. Nevertheless, we recognise these mechanisms as a promising future direction and discuss them in **Section 3 Discussion: Lines 434 - 452**. These are the newly added references (numbered as in the revised manuscript):

[12] Sapkota, R., Roumeliotis, K. I. & Karkee, M. Ai agents vs. agentic ai: A con ceptual taxonomy, applications and challenges. Information Fusion 126, 103599 (2026).

[63] Qian, C. et al. Chatdev: Communicative agents for software development. arXiv preprint arXiv:2307.07924 (2023).

[64] Yang, Y. et al. Agentnet: Decentralized evolutionary coordination for llm-based multi-agent systems. arXiv preprint arXiv:2504.00587 (2025).

[65] Wu, Q. et al. Autogen: Enabling next-gen llm applications via multi-agent conversations. First Conference on Language Modeling (2024).

[66] CrewAI. Crewai: The leading multi-agent platform. https://www.crewai.com/. Accessed: 2025-09-01.

[67] Li, H. et al. Advancing collaborative debates with role differentiation through multi-agent reinforcement learning. Proceedings of the 63rd Annual Meeting of the Association for Computational Linguistics (Volume 1: Long Papers), 22655–22666 (2025).

[68] Lu, S., Shao, J., Luo, B. & Lin, T. Morphagent: Empowering agents through self-evolving profiles and decentralized collaboration. arXiv preprint arXiv:2410.15048 (2024). [69] Raza, S., Sapkota, R., Karkee, M. & Emmanouilidis, C. Trism for agentic ai: A review of trust, risk, and security management in llm-based agentic multi-agent systems. arXiv preprint arXiv:2506.04133 (2025).

R2.4: There is no mechanism for the agents to simulate tasks, explore solution spaces, or verify strategies before execution which is a key requirement for high-assurance applications and agentic behavior.

Response: Thank you for your comment.

Such mechanisms can indeed reduce errors in the generated results and may improve efficiency by avoiding redundant tool or agent selections. However, the aim of our work is to demonstrate that multi-agent AI systems like PhenoAssistant can streamline computational workflows for plant phenotyping, rather than to exhaustively incorporate every possible solution at this stage.

Currently, PhenoAssistant addresses these issues through user supervision, which was deemed extremely important by the plant phenotyping researchers (our target users) we queried (as shown in Fig. 1 the user "Approval or correction" paths). They strongly advocated for a human-in-the-loop approach as a first step before full automation. Every step requires user approval before execution. While this verification mechanism prevents unmonitored errors, it does place higher demands on users' understanding of AI and computational tools, potentially increasing their workload. PhenoAssistant also allows users to save intermediate outputs (Fig. 1 the "Outputs" part) so that results can be checked and validated independently. Given that our target users possess strong domain expertise, this manual verification remains feasible.

However, your comment inspired us to implement a Critic agent which reviews the Manager agent's responses and help improve them. We conducted this new experiment when evaluating PhenoAssistant's tool selection ability and we found that adding such strategy verification mechanism can consistently improve its performance.

We discussed the results in **Section 2.3 Evaluation of PhenoAssistant: Lines 314 – 326, Table 1 (the "Manager + Critic" row), and Lines 354 – 355**. The detailed task-by-task result comparisons between the Manager-only and Manager + Critic design are provided at **Supplementary Table 2**. The system prompt of the Critic agent is provided at **Supplementary Note 5**.

Please note that, when addressing other reviewers' comments, the evaluation approach in tool selection has changed from manual inspection to using an LLM-based evaluator (R1.1), and the number of evaluation tasks has been increased from 10 to 20 (R1.1, R3.3). For more contexts, please refer to **Lines 283 – 313** and **Supplementary Note 4.1.**

We have also discussed to include other strategy verification mechanisms as future directions at **Lines 374 – 379**, with these references added (numbered as in the revised manuscript):

References:

[46] Golechha, S. & Garriga-Alonso, A. Among us: A sandbox for measuring and detecting agentic deception. arXiv preprint arXiv:2504.04072 (2025).

[47] Sapkota, R., Roumeliotis, K. I. & Karkee, M. Vibe coding vs. agentic coding: Fundamentals and practical implications of agentic ai. arXiv preprint arXiv:2505.19443 (2025).726

R2.5: Introduce agent-based simulation tools where agents can virtually test phenotyping hypotheses (e.g., nutrient deficiency responses(this is just an example, you can consider relevant example to your dataset but the example should be applied) before deploying real-world models

Response: Thank you for your comment.

We agree that pre-deployment simulation may reduce cost and risk in phenotyping experiments. Our contribution, however, is scoped to phenotype extraction and downstream analysis, which happen after the real-world plant experiments have already been conducted. Consequently, agent-based simulation falls outside the scope of our work.

While agent-based simulation is an active research area (e.g. in economic systems [55, 56] and physical domains [57, 58]), to our knowledge, mature simulators for plant phenotyping are not yet available. Recent work [59] also highlights robustness and efficiency challenges in agent-based simulation. Accordingly, integrating a well performed agent-based simulation mechanism for plants would require a dedicated research effort (e.g. a realistic plant growth model is required) rather than a short-term revision to our work.

Nevertheless, we think agent-based simulation is a promising future to extend PhenoAssistant's scope, and we have added **Lines 414 – 424 in Section 3 Discussion**. These are the newly added references when writing this discussion (numbered as in the revised manuscript):

[55] Geerling, W., Mateer, G. D., Wooten, J. & Damodaran, N. Chatgpt has aced the test of understanding in college economics: Now what? The American Economist 68, 233–245 (2023)

[56] Chen, Y., Liu, T. X., Shan, Y. & Zhong, S. The emergence of economic rationality of gpt. Proceedings of the National Academy of Sciences 120, e2316205120 (2023).731

[57] Shah, D., Osi´nski, B., Levine, S. et al. Lm-nav: Robotic navigation with large pre-trained models of language, vision, and action. Conference on robot learning, 492–504 (PMLR, 2023)

[58] Cui, C., Ma, Y., Cao, X., Ye, W. & Wang, Z. Drive as you speak: Enabling human-like interaction with large language models in autonomous vehicles. Proceedings of the IEEE/CVF Winter Conference on Applications of Computer Vision, 902–909 (2024).

[59] Gao, C. et al. Large language models empowered agent-based modeling and simulation: A survey and perspectives. Humanities and Social Sciences Communications 11, 1–24 (2024)..

R2.6: Try to integrate causal modeling agents that reason about cause-effect relationships in plant physiology and validate hypotheses using both statistical and symbolic methods.

Response: Thank you for your suggestion.

We agree that proper causal modelling method in plant physiology can help formulate mechanistic hypotheses and predict phenotypic responses under novel conditions. However, integrating such functionality would require substantial experimental design, data collection, and extensive validation in real-world settings, which was not feasible within the revision period. Similar to agent-based simulation (R2.5), causal modelling agents are actively explored but remain at an early stage, and are not yet readily available for direct deployment in plant phenotyping workflows. Nevertheless, with the rapid development of AI agents and agentic AI, we believe that agent-based causal modelling will become increasingly accessible in the future. To discuss this, we have added **Lines 425 - 433 in Section 3 Discussion**.

These are the newly added references (numbered as in the revised manuscript):

[12] Sapkota, R., Roumeliotis, K. I. & Karkee, M. Ai agents vs. agentic ai: A conceptual taxonomy, applications and challenges. Information Fusion 126, 103599 (2026).

[60] Han, K., Kuang, K., Zhao, Z., Ye, J. & Wu, F. Causal agent based on large language model. arXiv preprint arXiv:2408.06849 (2024).

[61] Wang, X. et al. Causal-copilot: An autonomous causal analysis agent. arXivpreprint arXiv:2504.13263 (2025).

[62] Chi, H. et al. Unveiling causal reasoning in large language models: Reality or mirage? Advances in Neural Information Processing Systems 37, 96640–96670 (2024).

R2.7: Write more discussion on Agentic AI how will it be different than a normal AI Agent, present some recent findings and literatures how they define this both terms and also write the discussion as Scalable Infrastructure and Governance in the future discussion part.

Response: Thank you for your suggestion.

We have included the definitions and comparison of agentic AI and AI agents in the Introduction section as mentioned at the response for R2.3.

We have discussed scalable infrastructure and governance in **Section 3 Discussion: Lines 453 – 475**. These are the newly added references (numbered as in the revised manuscript):

[12] Sapkota, R., Roumeliotis, K. I. & Karkee, M. Ai agents vs. agentic ai: A conceptual taxonomy, applications and challenges. Information Fusion 126, 103599 (2026).

[69] Raza, S., Sapkota, R., Karkee, M. & Emmanouilidis, C. Trism for agentic ai: A review of trust, risk, and security management in llm-based agentic multi-agent systems. arXiv preprint arXiv:2506.04133 (2025)

[70] Google Cloud. Announcing the agent2agent protocol (a2a). https://developers.googleblog.com/en/a2a-a-new-era-of-agent-interoperability/. Accessed: 2025-09-01.

Reviewer #2 (Remarks on code availability):

I will review full code during the revision as I have advised some experimentation revisions too which would need to update the code as well.

Response: Thank you for your thoughtful review. Our code repository is available at https://github.com/fengchen025/PhenoAssistant/ (latest version: branch v2). It contains implementation of PhenoAssistant as well as chat logs of case studies and evaluations.

Reviewer #3 (Remarks to the Author):

R3.0: Overall, this is an interesting and timely work that leverages the new wave of Agentic AI pipelines for automate plant phenotyping tasks. However, the main concerns are: (1) evaluation does not seem comprehensive, and (2) unclear how it may scale to add new tools and models to the toolkit. Hence, the claim seems a bit overblown.

Below are some detailed comments and suggestions.

Response: We thank you for recognising the novelty and timeliness of our work. We also appreciate your constructive concerns regarding the comprehensiveness of the evaluation and the scalability of our system. In this revision, we have made substantial efforts to address your comments, including the points mentioned above. Please see our detailed responses below.

R3.1: In Figure 4, i.e., case study 3, what framework is being used to make the models? For example, how is the appropriate model size selected?

Response: Thank you for your comment.

The model training function is implemented using PyTorch and Transformers (from Hugging Face). For new image classification tasks (Case Study 3), the system always finetunes DINOv2-base [75] which is a generalist vision foundation model, as described in *Section 4.2: Details of PhenoAssistant – Automatic Vision Model Training* (previously Section 7.2). Our previous work [74] has demonstrated that DINOv2-base is highly adaptable across various plant-related tasks.

At the current version of PhenoAssistant, we do not vary the model size, i.e. using a smaller model (DINOv2-small) or larger model (DINOv2-large), as the primary goal of our study is to demonstrate that model finetuning is a way to extend the capabilities of agent systems. **Considerations such as resource efficiency and model size selection are secondary in this context.** However, we do integrate both LoRA [39] and full-parameter finetuning options to allow users adapt to their hardware (e.g. GPUs), dataset size, and requirements for model performance, as described at **Section 4.2 Lines 562 - 569 (previously Section 7.2 Lines 324 - 331)**.

References (numbered as in the revised manuscript):

[39] Hu, E. J. et al. Lora: Low-rank adaptation of large language models. International Conference on Learning Representations 1, 3 (2022).811

[74] Chen, F., Giuffrida, M. V. & Tsaftaris, S. A. Adapting vision foundation models for plant phenotyping. Proceedings of the IEEE/CVF International Conference on Computer Vision Workshops, 604–613 (2023)

[75] Oquab, M. et al. Dinov2: Learning robust visual features without supervision. arXiv preprint arXiv:2304.07193 (2023)

R3.2: L218: It seems it is only predicting the type of model to select (e.g., instance segmentation, image classification, etc.), but which exact model is selected from the model zoo is not being evaluated.

Response: Thank you for your comment.

At L218 of our original manuscript, we introduced the evaluation of vision model selection. Its goal is to assess whether PhenoAssistant can recommend the appropriate type of computer vision model for a given phenotyping task. This ensures that, when the existing model zoo does not contain a suitable model, PhenoAssistant can still guide the user to prepare labelled datasets correctly for automatic model training. That is why the evaluation only asks PhenoAssistant to predict the type of model rather than identifying a specific model instance.

We did not evaluate which exact vision model from the model zoo is selected because each model is uniquely identified by our naming convention, which is described at **Section 4.2 Lines 530 – 532 (previously Section 7.2 Lines 292 - 294)**: "...Each vision model is uniquely identified using the naming convention: {plant-species}_{task}_{(optional) training-dataset}_{model}_{(optional) finetuning-method}..."

For example, the models demonstrated in Case Study 1 & 2 are named as below (also stored at our code repository at model_zoo.json):

(i) Case Study 1: "arabidopsis_leaf-instance-segmentation_cvppp2017-a1a4_m2fb_fullft". "cvppp2017-a1a4" indicates the training dataset, "m2fb" indicates the model architecture (Mask2Former [71]), and "fullft" indicates full-parameter finetuning.

(ii) Case Study 2: "potato_leaf-instance-segmentation_leaf-only-sam". "leaf-only-sam" is the model architecture Leaf Only SAM [40].

As both the plant species and the task are encoded in the identifier, PhenoAssistant only needs to match the correct model given users' description.

References (numbered as in the revised manuscript):

[40] Williams, D., Macfarlane, F. & Britten, A. Leaf only sam: A segment anything pipeline for zero-shot automated leaf segmentation. Smart Agricultural Technology 8, 100515 (2024).

[71] Cheng, B., Misra, I., Schwing, A. G., Kirillov, A. & Girdhar, R. Masked-attention mask transformer for universal image segmentation. Proceedings of the IEEE/CVF Conference on Computer Vision and Pattern Recognition,1290–1299 (2022).

R3.3: The granularity of prediction in invasion model selection and the number of tool selections (currently 10) and data analysis tasks (currently 10 as well) should be evaluated.

Response: Thank you for your suggestion.

We interpret "granularity of prediction in invasion model selection" as the level of detail in vision model selection. As noted in our response to R3.2, we do not evaluate the choice of exact models, since our naming convention for models in the model zoo has encoded plant species and task.

For the number of tool selection tasks:

In this revision, we have expanded the number of tasks to evaluate tool selection from 10 to 20. This expansion included more plant species, trait types, and analysis requests.

We discussed the evaluation process and results at **Lines 283 – 313** and **Table 1 (the "Manager" row)**. Please note that when addressing other reviewers' comments (R1.1), we have replaced the original human inspection method (i.e. to tell if the generated tool chains from PhenoAssistant is correct or not) to an LLM-based evaluator.

The newly added task descriptions and detailed per-task evaluation results are provided at **Supplementary Note 4.1 Tasks 11 – 20**, and **Supplementary Table 2 (under the "Manager" column)**.

For the number of data analysis tasks:

Similarly, we expanded the data analysis evaluation task set from 10 to 20 tasks. This expansion introduced four new categories, namely Analysing Plots (Tasks 11–14), Converting File Formats (Tasks 15–16), Computing New Metrics (Task 19), and Analysing New Metrics (Task 20), as well as additional tasks within the existing category Computing Statistics (Tasks 17–18) that was previously less represented.

After this expansion, accuracy on data analysis tasks decreased from 100% (10/10) to 85% (17/20). The three errors all occurred in the newly added Analysing Plots category. We have discussed this expanded evaluation on data analysis at **Lines 341 – 351**. The prompts for the newly added tasks are provided at **Supplementary Note 4.3 Tasks 11 – 20**.

R3.4: L254: The accuracy of reproduced pipelines should also be given.

Response: Thank you for your suggestion.

We prompted PhenoAssistant to extract and re-apply the pipeline generated at *Case Study 1 Task 1* five times on the same input file.

Each run produced a new CSV output file. We compared both the extracted pipelines (i.e. Python codes) and the corresponding outputs. We found that although the extracted pipelines differed slightly in structure across runs, all were executable by PhenoAssistant and consistently generated identical outputs.

We revised **Supplementary Note 3** to include this experiment at **Lines 41 - 46**. The experimental process has also been recorded in our code repository (branch v2, case1_pipe.ipynb).

R3.5: L304: It is rightly noted that it is practically impossible to pre-integrate every required vision model. To solve this problem, a mechanism for dynamically fetching onion models could help. The publicly available open-sourced models with the 'agriculture' tag, for example, could be fetched from Kaggle. https://www.kaggle.com/models?query=agriculture&owner-type=user

Response: Thank you for your suggestion.

We interpret the phrase "dynamically fetching onion models" to mean that, given user-provided task descriptions (including both the plant species and the intended phenotyping task), PhenoAssistant could dynamically retrieve the most suitable open-sourced model and execute it to address the user's requests.

However, after carefully investigating open-sourced models on AI community platforms including Kaggle and HuggingFace, we identified several drawbacks that currently prevent us from integrating such a mechanism:

(i) Model identifiers and model cards on these platforms are not standardised and often do not provide sufficient information for an LLM agent to reliably select models according to the provided plant species and tasks. For example:

a. https://www.kaggle.com/models/abhisheksawkar/resnet-50-for-agriculture: The model is named “RESNET 50 FOR AGRICULTURE”, but a model description is not provided in the model card.

b. https://huggingface.co/linkanjarad/mobilenet_v2_1.0_224-plant-disease-identification?inference_provider=hf-inference: The model is named “mobilenet_v2_1.0_224-plant-disease-identification” with the name of the training dataset provided in the model card, but it remains difficult to directly infer which plant species it covers and what specific diseases it can detect.

(ii) Even when models provide more comprehensive descriptions to infer the plant species and tasks (e.g. https://huggingface.co/wambugu71/crop_leaf_diseases_vit?inference_provider=hf-inference), an additional mechanism is still required to determine whether user-provided data falls outside the distribution of the training data. For instance, user data may have been captured under different lighting conditions or with a different camera than the training data, which could affect the performance of the selected model. This also requires model providers to supply clearer descriptions of the source of their training datasets and make them publicly available.

(iii) Another challenge is the reliability of open-sourced model repositories themselves. External repositories may change their access requirements, alter input/output formats, or even remove models altogether, making it difficult to ensure consistent integration.

(iv) Moreover, automatically incorporating external models poses security risks that we aim to avoid in plant phenotyping research. For example, unvetted models could contain malicious components or adversarial behaviours.

For these reasons, we encourage potential users to rely on the integrated model training function (Case Study 3) to train new computer vision models on their own data.

Nevertheless, we recognise that dynamically fetching open-sourced models has great potential to extend PhenoAssistant’s vision capabilities. We have discussed the above as future work and challenges in **Section 3 Discussion: Lines 388 - 407**.

In addition, we have implemented a functionality to allow users to use models that support HuggingFace Inference API, but they need to know the model identifiers and the expected input/output formats of the models. This is documented in our code repository v2 branch.

R3.6: L324: Where is the use of LoRA fine-tuning demonstrated in the presented use cases?

Response: Thank you for your comment.

At L324 in our original manuscript, we mention "PhenoAssistant supports two fine-tuning strategies: low-rank adaptation (LoRA) and full fine-tuning, ..."

We apologised that it was previously not clearly presented in Case Study 3. We have now revised **Fig.7 and its caption (highlighted in orange)**, and **Lines 265 - 266** to explicitly indicate that users can instruct PhenoAssistant to choose between LoRA and full-parameter finetuning after uploading a dataset.

An example was also supplied at our code repository at branch v2, case3.ipynb (a screen shot is presented below):

```
--------------------------------------------------------------------------------
manager (to Admin):

I provide the following finetuning methods for image classification models:

1. **LoRA (Low-Rank Adaptation)**: This method adapts the pre-trained model by learning low-rank updates, which is computatio
nally efficient and requires less memory.

2. **Full Fine-Tuning (FullFT)**: This method involves fine-tuning all the parameters of the pre-trained model, which can be
more computationally intensive but may yield better performance for some tasks.

Please let me know which finetuning method you would like to use, and I will proceed with the training.

--------------------------------------------------------------------------------
Admin (to manager):

let's do lora

--------------------------------------------------------------------------------
manager (to Admin):

***** Suggested tool call (call_5x6lqzDLk2aBPBDZjvz8DiyA): finetune_image_classification *****
Arguments:
{"dataset_name":"winter-wheat_nutri-defi-identify_dndww20","ft_method":"lora","train_bs":32,"val_bs":1,"img_w":512,"img_h":51
2,"lr":0.0001,"epochs":1}
**********************************************************************************************

--------------------------------------------------------------------------------

>>>>>>>> NO HUMAN INPUT RECEIVED.

>>>>>>>> USING AUTO REPLY...

>>>>>>>> EXECUTING FUNCTION finetune_image_classification...
Model 'winter-wheat_nutri-defi-identify_dndww20_dino2b_lora' does not exist. Proceed to train the model.
Generating train split: 100%|██████████| 1332/1332 [00:08<00:00, 154.64 examples/s]
Generating test split: 100%|██████████| 468/468 [00:03<00:00, 138.97 examples/s]
```

R3.7: L580 - L635: The pipeline is supposed to predict one of these labels: 'instance segmentation, image classification, and image regression,' but the inputs themselves give it away. For example, the inputs were: Flower color classification, Leaf disease segmentation, Fruit ripening stage classification. So, even though it is reported that this evaluation is pretty surface level, the reported performance of 98% does not mean much.

Response: Thank you for your comment. We appreciate your observation that some of the original task descriptions may have revealed the expected vision model type.

In this revision, we have substantially updated the list of evaluation tasks for vision model selection. Specifically, we reworded all tasks to avoid directly referencing vision model types including "classification," "segmentation," and "detection". We also increased linguistic diversity of the tasks by expressing them in varied forms, such as questions (e.g. "What type of nutrient deficiency is present in these plants? ", "Are these plants healthy or diseased? ") and full instructions (e.g. "Highlight the weeds in these field images", "Delineate the crop rows in these images"), rather than presenting short phrases only. In addition, we clarified some previously ambiguous tasks (e.g. changing "plant vigor scoring" to "plant vigor scoring on a continuous scale of 0 to 1").

These updates can be found in **Supplementary Note 4.2**, where we highlight the changed tasks and report PhenoAssistant's predicted model types for each task. PhenoAssistant achieved 100% accuracy in the new evaluation (previously 98%), which is now discussed at **Lines 327 – 338.**

This performance suggests that, with a well-designed system prompt, GPT-4o (as the manager LLM of PhenoAssistant) can interpret varied natural-language task descriptions for plant phenotyping and map them to the appropriate vision model type. Moreover, clearer task descriptions help correct predictions. For example, rephrasing the "plant vigor scoring" to "plant vigor scoring on a continuous scale of 0 to 1" enabled the system to correctly infer

that image regression was the appropriate choice, rather than image classification as previously predicted.

R3.8: How is the model going to know if the image being asked for is trained for it? VLMs can take general input, but these specialized trained models cannot, so how are they fed? For example, if any image from another dataset is captured which does not correspond to training data, then it is a known limitation with traditional models.

Response: Thank you for your comment.

As noted in our response to R3.2, PhenoAssistant automatically selects the most suitable vision model by matching the model identifier with the user-specified task descriptions (e.g. plant species, tasks, and where available, the training dataset name). If the task descriptions also include information about the source of the user-provided data, it will allow PhenoAssistant to better select models trained on the same data distribution.

However, we did not implement an automatic mechanism to verify whether user-provided data are compatible (i.e. in-distribution) with the selected model. At present, this assessment should be made by the users themselves, for example inspecting the saved intermediate outputs. To support this, PhenoAssistant allows users to save intermediate outputs (Fig. 1 the "Outputs" part) such as vision models' outputs and the extracted phenotypes. Since our target users are plant phenotyping researchers and practitioners, this manual verification is reasonable, as they possess the expertise to assess whether the extracted phenotypes are reliable. In addition, users can leverage the integrated model training function (e.g. if they are not satisfied with the extracted phenotypes) to train a new model on their own dataset, thereby ensuring the data-model compatibility.

Nevertheless, we really appreciate your comment and have indicated this limitation and its potential solutions, e.g. integrating out-of-distribution (OOD) detection mechanisms [48, 49] or plant and agricultural vision foundation models [50, 51], in **Section 3 Discussion: Lines 380 - 387**. It is worth noting that reliable plant and agricultural vision foundation models were largely unavailable at the time our study was conducted.

We have added these references (numbered as in the revised manuscript):

[48] Averly, R. & Chao, W.-L. Unified out-of-distribution detection: A model-specific perspective. Proceedings of the IEEE/CVF International Conference on Computer Vision, 1453–1463 (2023).

[49] Miyai, A. et al. Generalized out-of-distribution detection and beyond in vision language model era: A survey. arXiv preprint arXiv:2407.21794 (2024).

[50] Tonmoy, M. R., Hossain, M. M., Dey, N. & Mridha, M. Mobileplantvit: A mobile-friendly hybrid vit for generalized plant disease image classification. ArXiv preprint arXiv:2503.16628 (2025).

[51] Han, B. et al. Fomo4wheat: Toward reliable crop vision foundation models with globally curated data. arXiv preprint arXiv:2509.06907 (2025).

R3.9: Make a clear distinction between a module and an agent. For example, why is statistical test listed as an agent?

Response: Thank you for pointing out this area of confusion and your helpful suggestion.

We categorised the tools of PhenoAssistant by their objectives, i.e. phenotype extraction and phenotype analysis to suit the interests of a wider audience of *Nature Communications* (e.g. plant and agricultural researchers who are not familiar with LLMs and LLM-based agent systems). Within phenotype analysis, most tools are implemented as LLM agents, while statistical tests are implemented as dedicated modules. We have revised the manuscript to be clearer:

(i) We now introduce statistical tests before LLM agents and explicitly state that statistical tests are implemented as dedicated modules. Please see **Lines 180 – 184**.

(ii) **Fig. 1** has been updated to rename "Statistical Test" to "**Statistical Test Modules**" and to reposition it above the LLM agents under "Phenotype analysis".

R3.10: Model Context Protocol (MCP) is a standardized way to connect to external data sources and tools through a unified interface. Instead of PhenoAssistant creating its own custom toolkit integration system, they could have used MCP to connect their LLM to the vision models, statistical tools, etc.

Response: Thank you for your comment.

MCP was introduced on November 25, 2024 (according to https://en.wikipedia.org/wiki/Model_Context_Protocol) as a standardised method to connect data and tools with LLMs.

However, we began designing and building the framework of PhenoAssistant in May 2024, and by the time MCP was introduced, we had implemented most of its functions.

As evidence of this timeline, we refer to the earlier version of PhenoAssistant presented at the Computer Vision in Plant Phenotyping and Agriculture Workshop 2024 (CVPPA 2024), where we published an abstract titled "PhenoGPT: Towards Language Interaction with Vision Models for Plant Phenotyping" (https://cvppa2024.github.io/proceedings/). This abstract was submitted in July 2024 to meet the workshop's submission deadline.

We also presented a demo of PhenoAssistant to the intended users at the UK Phenomics Town Hall and Conference 2024, which was held at 4 and 5 September, 2024 (https://phenomuk.org/conference/) and received highly positive feedbacks.

More importantly, the main goal of our work is to demonstrate that agent systems can help plant phenotyping workflow generation and execution; the specific method of tool integration does not affect the demonstration of this goal.

Nevertheless, we really appreciate your comment and recognise that MCP is a promising protocol for future development of PhenoAssistant. Although it is new, as the ecosystem of MCP-compatible tools grows, particularly when more plant-related resources emerge, it could become more valuable to extend PhenoAssistant and other similar systems. We include the discussion of MCP in **Section 3 Discussion: Lines 408 – 413**.

R3.11: Chat logs and evaluation results are provided in a public repository, but the software/tool itself is not available. Also, it would help to publicly document the method to add new agents in the toolkit, after making it publicly accessible.

Response: Thank you for your comment and suggestion.

The source code of PhenoAssistant, including the agents and the integrated tools, has been included in our Github repository at https://github.com/fengchen025/PhenoAssistant (previously the "main" branch, now the "v1" branch) since our first submission; a detailed README file to guide users to properly configure and run PhenoAssistant was also provided.

We have now extended the documentation to include instructions on how to add new tools and agents. This guidance is available in the README file under the "v2" branch. We really appreciate your feedback, which helps us make PhenoAssistant more accessible.

R3.12: A matrix table containing the feature comparison of PhenoAssistant with other existing tools would be a great addition. Some tools available online in this space are: https://github.com/Taranshah9/MumbaiHacks2024_LlamaMia

https://github.com/nalinrajendran/eco-agri-bot

https://github.com/jayeshbhandarkar/AgriGenius

Response: Thank you for your suggestion.

We have added **Supplementary Note 1** to compare the code availability, primary scope, key ability, system type, and whether image inputs are allowed between our PhenoAssistant and 10 other LLMs or LLM-based agent systems for plant science and agriculture (including the second and third examples you supplied). We also added **a footnote at page 3** to cross-reference this supplementary information.

Please note that we were unable to include the first example you provided (LlamaMia) as the URL was invalid.

Reviewer #4 (Remarks to the Author):

I co-reviewed this manuscript with one of the reviewers who provided the listed reports. This is part of the Nature Communications initiative to facilitate training in peer review and to provide appropriate recognition for Early Career Researchers who co-review manuscripts.

Reviewer #4 (Remarks on code availability):

I co-reviewed this manuscript with one of the reviewers who provided the listed reports. This is part of the Nature Communications initiative to facilitate training in peer review and to provide appropriate recognition for Early Career Researchers who co-review manuscripts.

Response: We sincerely thank you for your thoughtful review on both our manuscript and code repository. Our code repository is available at https://github.com/fengchen025/PhenoAssistant/ (latest version: branch v2), which includes implementation of PhenoAssistant, together with the chat logs of case studies and evaluations.

Responses to Reviewers' Comments

To All Reviewers:

We sincerely thank you for your time and effort in reviewing our revised manuscript and response letter. We greatly appreciate the constructive comments you provided, which have helped us to further improve our work. We have addressed all concerns, as detailed in our point-by-point responses below.

Please note that all revisions made in the manuscript and supplementary information are highlighted in orange. For better maintenance, we have also transferred our code repository to our organisation's Github page at https://github.com/vios-s/PhenoAssistant/.

Reviewer #1 (Remarks to the Author):

I am pleased with the extensive response and also the changes made to the manuscript based on my comments. I have no further comments

Response: We are pleased that our revisions have addressed your concerns and appreciate the time and effort you have devoted to improving our manuscript.

Reviewer #2 (Remarks to the Author):

Great Revision. However, there are still spaces to improve the paper with minor and important changes

R2.1: In the abstract, a proper GitHub repository link could be added as the last sentence.

Response: Thank you for your suggestion.

We have now added the repository link to Abstract (Lines 46 – 47).

R2.2: Figure 2, 3 and 4 caption could be more detailed for better read(basic to advanced reader audience should be focused for captioning such figures)

Response: Thank you for your suggestion.

Following your input, we have now detailed the captions of Figure 2, 3, and 4 to make them more comprehensive and to improve readability.

R2.3: Figure 5, the leaf count, plant diameter and plant perimeter graphs are hard to see and understand even with glasses. Please enlarge all those graphs to PLA size graph at least for better visibility

Response: Thank you for your suggestion.

The current layout of Figure 5 already occupies an entire page in the main text, making it difficult to further enlarge the plots within the figure. To address this concern, we have opted to add a new section to Supplementary Information (Supplementary Note 4) to include the enlarged versions of the PLA, leaf count, plant diameter, and plant perimeter plots. We have also added "Enlarged versions of the saved plots in Task 2 are provided in Supplementary Note 4" at the end of the caption of Figure 5 to direct readers to the enlarged plots.

Reviewer #2 (Remarks on code availability):

You could add some images of manuscript in the github page too, maintain page like a blog kind of information too with images that shows a proper instruction about the paper....

Response: Thank you for your suggestion.

We have now included a brief introduction of our paper (see below), along with Figure 1 (Design of PhenoAssistant), Figure 5 (Case Study 1) and Figure 6 (Case Study 2) as examples demonstrating PhenoAssistant's capabilities, in our code repository https://github.com/vios-s/PhenoAssistant/ (branch v2).

The introduction:

"PhenoAssistant is a multi-agent AI system designed to streamline complex plant phenotyping workflows through natural language interaction. It integrates a central manager agent with a specialised toolkit that combines modules (e.g., an extendable vision model zoo) and other LLM agents with specific roles (e.g., coding and data visualisation). Together, these components enable phenotype extraction and the subsequent data analysis tasks including statistical testing, plot creating and pipeline reproducing, allowing users to conduct image-based plant phenotyping with less technical effort."

Reviewer #3 (Remarks to the Author):

Here are my feedback on the authors' responses to my questions.

R3.1: In Figure 4, i.e., case study 3, what framework is being used to make the models? For example, how is the appropriate model size selected?
Response Assessment:
– Satisfied/Unsatisfied: Satisfied
– Additional Comments: I agree the model size and optimizations become secondary in this context. The authors say ”we do integrate both LoRA [39] and full-parameter finetuning options to allow users adapt to their hardware (e.g. GPUs), dataset size, and requirements for model performance.” However, there is a caveat that it takes more effort on the logistical side of setting up everything on the computer, which could vary from one system to another, to use the full capability of the hardware.

Response: Thank you for your positive assessment of our revision and for your helpful additional comment.

We have now added a note to our code repository https://github.com/vios-s/PhenoAssistant/ (branch v2) under *Adding External Tools and Agents* to remind users of the potential logistical efforts of setting up their systems:

“You can also prompt PhenoAssistant to train new computer vision models on your own annotated data. Please refer to case3.ipynb for an example. PhenoAssistant supports both LoRA and full fine-tuning approaches. Be sure to check your GPU configuration and choose the method that best matches your computational resources and needs.”

R3.2: L218: It seems it is only predicting the type of model to select (e.g., instance segmentation, image classification, etc.), but which exact model is selected from the model zoo is not being evaluated.
Response Assessment:
– Satisfied/Unsatisfied: Unsatisfied
– Additional Comments: While I acknowledge the authors’ explanation that they evaluate model type prediction rather than exact model selection, this evaluation task is too simplistic to properly assess the framework’s capabilities. The high accuracy numbers reported don’t provide meaningful confidence in the system because the task of categorizing into broad model types (instance segmentation, image classification, etc.) is relatively easy. A more challenging evaluation would better demonstrate the system’s real-world utility.

Response: Thank you very much for your suggestion.

We have now extended our evaluation to include assessing the ability of PhenoAssistant to select specific models from the model zoo, given plant phenotyping tasks. This new evaluation is named as Vision Model Selection II, while the previous evaluation focusing on

recommending vision model types is now referred to as Vision Model Selection I.

Vision Model Selection II comprises 20 plant phenotyping tasks and a model zoo containing 30 model identifiers, each following the naming convention {plant-species}_{task}_{model} (i.e. the naming rule of PhenoAssistant's vision models as described in the Methods section). To make the evaluation more realistic and challenging, only 15 of these model identifiers can be matched with 15 of the 20 plant tasks, while the remaining 15 model identifiers act as distractors.

PhenoAssistant is prompted to match each plant task with the most suitable model identifier from the vision model zoo. It achieves 100% accuracy in this challenging evaluation. For the 15 tasks with corresponding model identifier, it successfully identifies the correct match. For the remaining 5 tasks that cannot be addressed by the provided models, it recognises that no suitable model exists and recommends an appropriate model type for fine-tuning. This evaluation shows PhenoAssistant's potential real-world utility for addressing diverse plant phenotyping tasks using an expanded vision model zoo.

We have discussed this new evaluation at Section 2.3 Lines 344 - 365. The prompt used in this evaluation and PhenoAssistant's response are provided in Supplementary Note 5.3.

R3.3: The granularity of prediction in invasion model selection and the number of tool selections (currently 10) and data analysis tasks (currently 10 as well) should be evaluated.
Response Assessment:
– Satisfied/Unsatisfied: Satisfied
– Additional Comments: I acknowledge that the authors added meaningful numbers and evaluation details for the current scope of their toolkit. However, this does not fully address the scalability issue since the process remains manual. For the scope of this paper, the evaluation is adequate, but this manual limitation should be noted as a caveat for future scalability.

Response: Thank you for your positive assessment of our revision and for your helpful additional comment.

We have now noted the manual involvement in the evaluation process as a limitation and discussed it as part of future work in the Discussion section (Lines 404–408).

R3.4: L254: The accuracy of reproduced pipelines should also be given.
Response Assessment:
– Satisfied/Unsatisfied: Indifferent

– Additional Comments: Case study 1 provides anecdotal evidence that sparks confidence, but reproduction is limited to one case only.

Response: Thank you for your comment.

In this revision, we have further evaluated the pipeline reproduction function on these additional tasks:

1. **Case Study 1 Task 2 (compute and plot statistics of Arabidopsis phenotypes)**. PhenoAssistant's tool calls for this task involve:
    a. *Table Analyser Agent*, which computes and saves the mean and standard deviation of phenotypes.
    b. *Data Visualiser Agent*, which generates and saves plots of the computed statistics.
2. **Case Study 1 Task 4 (perform statistical tests on Arabidopsis phenotypes)**. PhenoAssistant's tool calls for this task include two *Statistical Test Modules* to perform ANOVA and Tukey-Kramer tests, respectively.
3. **Case Study 2 Task 1 (extract and save potato phenotypes)**. PhenoAssistant's tool calls for this task include:
    a. *Instance Segmentation Module*, which performs leaf instance segmentation on images.
    b. *Phenotype Computation Module*, which extracts relevant leaf phenotypes from the instance segmentation results.
    c. *Code Writer Agent*, which merges the computed phenotypes with metadata and saves them into a CSV file.

Following the same procedure used previously for **Case Study 1 Task 1**, PhenoAssistant was instructed to extract and reapply the generated pipeline for each of the above tasks 5 times using the same input file. We then evaluated both the extracted pipeline (Python code) and the results of each reproduction.

As with our earlier findings, the extracted pipelines for each task varied in content and structure due to inherent LLM stochasticity. In terms of execution and produced results, all tasks achieved a high success rate:

- **Case Study 1 Task 2** was successfully executed and produced correct results in 4/5 runs. One run failed due to the extracted pipeline cannot be executed.
- All other tasks were successfully executed and produced correct results in all 5 runs.

Including our previously reported results on **Case Study 1 Task 1** (which is similar to **Case Study 2 Task 1** but applied to Arabidopsis using a different segmentation model), we have now evaluated pipeline reproduction across four distinct tasks, covering two species and multiple phenotype analysis purposes. More importantly, these tasks covered representative pipeline structures, including mixed module–agent sequences (**Case Study 1 Task 1**, **Case Study 2 Task 1**), pure agent-based sequences (**Case Study 1 Task 2**), and pure module-based sequences (**Case Study 1 Task 4**).

We believe that our evaluations have demonstrated the generalisability and robustness of PhenoAssistant's pipeline reproduction capability.

We have discussed the evaluation of pipeline reproduction at Supplementary Note 3 (Lines 44 – 68).

R3.5: L304: It is rightly noted that it is practically impossible to pre-integrate every required vision model. To solve this problem, a mechanism for dynamically fetching onion models could help. The publicly available open-sourced models with the 'agriculture' tag, for example, could be fetched from Kaggle.
https://www.kaggle.com/models?query=agriculture&owner-type=user
Response Assessment:
– Satisfied/Unsatisfied: Satisfied
– Additional Comments: The authors rightly noted the limitations of fetching online models and thus pre-fetching is indeed necessary in this context to add reliability.

Response: Thank you for your feedback. We are glad that our revision has fully addressed your concern.

R3.6: L324: Where is the use of LoRA fine-tuning demonstrated in the presented use cases?
Response Assessment:
– Satisfied/Unsatisfied: Satisfied
– Additional Comments: It has now been added in revision.

Response: Thank you for your feedback. We are glad that our revision has fully addressed your concern.

R3.7: L580 - L635: The pipeline is supposed to predict one of these labels: 'instance segmentation, image classification, and image regression,' but the inputs themselves give it away. For example, the inputs were: Flower color classification, Leaf disease segmentation, Fruit ripening stage classification. So, even though it is reported that this evaluation is pretty

surface level, the reported performance of 98% does not mean much.
Response Assessment:
– Satisfied/Unsatisfied: Satisfied
– Additional Comments: The list of evaluation tasks has been diversified. However, the question of using this as a metric to validate the framework is still questionable (as shared above). But I am satisfied with the concern of having revealing labels in the input.

Response: Thank you for your positive assessment of our revision and for your helpful additional comment.

As already noted in our response to R3.2, we have added new evaluation tasks to provide a more granular assessment of vision model selection. Together with the existing case studies and evaluations, we believe that we have reasonably and informatively demonstrated PhenoAssistant's capabilities, potential, and limitations.

R3.8: How is the model going to know if the image being asked for is trained for it? VLMs can take general input, but these specialized trained models cannot, so how are they fed? For example, if any image from another dataset is captured which does not correspond to training data, then it is a known limitation with traditional models.
Response Assessment:
– Satisfied/Unsatisfied: Indifferent
– Additional Comments: This is rightly noted as a limitation (out of distribution). However, the expectation that experts will be able to identify issues from intermediary outputs is not reasonable because images could be taken from different cameras and human eye/expert cursory evaluation might not catch such cases. This could be a low hanging fruit for further improvement. If the system could detect out of distribution cases, that would add hugely to the reliability of the system.

Response: Thank you for your comment.

We agree that incorporating an out-of-distribution (OOD) detection component into PhenoAssistant would be valuable and could further enhance the system's reliability by helping users identify when a selected model may not generalise well on the provided data. However, there is currently no established gold-standard approach for OOD detection in specialised computer vision models [1, 2]. Developing such functionality remains an open research challenge that requires substantial additional work. More importantly, as our study focuses on demonstrating how an LLM agent system can support plant phenotyping workflows, automatic OOD detection falls beyond our current scope.

We would also like to clarify that, as mentioned in our previous response, the suggestion for

users to inspect intermediate results refers to examining outputs such as segmentation masks and extracted phenotypes, rather than analysing the raw input images themselves. When the outputs are clearly incorrect (e.g. segmentation masks fail to focus on the target regions or extracted leaf count falls outside a reasonable range), users can readily recognise that the selected vision model is unsuitable for their data, without needing to know image capturing details such as camera specifications.

Nevertheless, inspired by your comment, we implemented and evaluated several OOD detection methods for both instance segmentation and image classification. To reflect the realistic setting of integrating external models into LLM-based agents (e.g., via APIs), we used methods that only require access to model inputs and outputs, without relying on any intermediate features.

Implemented and Evaluated OOD Detection Methods:

Let x be an input image, and let the model produce $M(x)$ predictions with confidence scores $\{s_j(x)\}_{j=1}^{M(x)}$. For instance segmentation, $M(x)$ denotes the number of instance masks and $s_j(x)$ is the confidence score of mask j. For image classification, $M(x)$ is equal to 1 and $s_j(x)$ is the confidence score of the predicted class. We define the image-level confidence score as

$$S(x) = \frac{1}{M(x)} \sum_{j=1}^{M(x)} s_j(x).$$

Given these definitions, the OOD detection methods we evaluated are defined as follows (note that all τ terms denote predefined threshold values):

1. **Score-based**: The input image is flagged as OOD if

$$S(x) < \tau_S.$$

2. **Perturbation-based**: Since a single inference may not be sufficiently robust for OOD detection, we also evaluate a perturbation-based approach inspired by [3, 4]. Specifically, for each image x, we generate K noisy variants $\{x_k\}_{k=1}^{K}$ by adding Gaussian noise. We then run the model on both the original image and all its noisy variants, and calculate the average image-level confidence score across all outputs:

$$\overline{S}(x) = \frac{1}{K+1} \sum_{k=0}^{K} S(x_k),$$

where $x_0 = x$ denotes the original image.

For image classification, we only use this score. The original image is flagged as OOD if

$$\overline{S}(x) < \tau_{sc}.$$

For instance segmentation, we additionally consider the variation $V(x)$ in the number of predicted instances as a measure of uncertainty in the model's outputs. We quantify this variation using the relative standard deviation:

$$V(x) = \frac{std(M(x_0),\ldots,M(x_K))}{mean(M(x_0),\ldots,M(x_K))+\varepsilon},$$

where ε is a small constant to avoid division by zero.

An image is flagged as OOD if

$$\overline{S}(x) < \tau_{si} \quad \text{or} \quad V(x) > \tau_{vi}.$$

3. **VLM-based (instance segmentation only)**: For instance segmentation, we also use a vision–language model (GPT-4o) to assess segmentation quality. Given the original image and its predicted masks, the VLM produces a quality score:

$$Q(x) \in [0,1],$$

where 0 indicates totally wrong segmentation and 1 indicates perfect segmentation.

The image is flagged as OOD if

$$Q(x) < \tau_q.$$

Experiments and Results:

We evaluated these methods on a leaf instance segmentation model (from Case Study 1) and an image classification model (from Case Study 3). For each model, we select 10 in-distribution (ID) images and 10 out-of-distribution (OOD) images, and report how many images are detected as OOD. Ideally, this metric should be 0/10 for ID images and 10/10 for OOD images. The selected thresholds and results are summarised below.

Image classification:

1. Score-based ($\tau_s = 0.5$): ID = 1/10, OOD = 5/10
2. Perturbation-based ($\tau_{sc} = 0.5$): ID = 2/10, OOD = 5/10

Instance segmentation:

1. Score-based ($\tau_s = 0.9$): ID = 0/10, OOD = 7/10
2. Perturbation-based ($\tau_{si} = 0.9,\ \tau_{vi} = 0.1$): ID = 1/10, OOD = 10/10
3. VLM-based:
   a. $\tau_q = 0.5$: ID = 0/10, OOD = 5/10

b. $\tau_q = 0.9$: ID = 0/10, OOD = 10/10

In summary, these input–output–based OOD detection methods offer some ability to distinguish ID and OOD samples. However, their performance depends on careful threshold optimisation, which requires manual tuning and limits generalisability. This suggests that more automated and robust OOD detection approaches are still needed, especially in the context of LLM-based agents. In the future, if reliable and universally applicable OOD detection methods become available, we will explore their integration into PhenoAssistant.

References:

[1] Lu S, Wang Y, Sheng L, He L, Zheng A, Liang J. Out-of-distribution detection: A task-oriented survey of recent advances. ACM Computing Surveys. 2025 Sep 8;58(2):1-39.

[2] Gawlikowski J, Tassi CR, Ali M, Lee J, Humt M, Feng J, Kruspe A, Triebel R, Jung P, Roscher R, Shahzad M. A survey of uncertainty in deep neural networks. Artificial Intelligence Review. 2023 Oct;56(Suppl 1):1513-89.

[3] Kendall A, Gal Y. What uncertainties do we need in bayesian deep learning for computer vision?. Advances in neural information processing systems. 2017;30.

[4] Zhang B, Zhu J, Wang Z, Liu T, Du B, Han B. What if the input is expanded in OOD detection?. Advances in Neural Information Processing Systems. 2024 Dec 16;37:21289-329.

R3.9: Draw a line between calling it a module and an agent. For example, why is statistical test listed as an agent
Response Assessment:
– Satisfied/Unsatisfied: Satisfied
– Additional Comments: Thanks for clarifying in text.

Response: Thank you for your feedback. We are glad that our revision has fully addressed your concern.

R3.10: Model Context Protocol (MCP) is a standardized way to connect to external data sources and tools through a unified interface. Instead of PhenoAssistant creating its own custom toolkit integration system, they could have used MCP to connect their LLM to the vision models, statistical tools, etc.
Response Assessment:
– Satisfied/Unsatisfied: Satisfied
– Additional Comments: The authors acknowledge MCP as a valid alternative but note their

custom integration was developed before MCP became widely adopted. While MCP would indeed reduce redundant integration code, the current system's modular design allows for migration to MCP in future versions.

The code is now public on GitHub along with v1 and v2 releases. Instructions to run the tool and add new agents are included. Chat logs and evaluation results are also available.

Response: Thank you for your feedback. We are glad that our revision has fully addressed your concern.

R3.11: A matrix table containing the feature comparison of PhenoAssistant with other existing tools would be a great addition. Some tools available online in this space are:

https://github.com/Taranshah9/MumbaiHacks2024_LlamaMia

https://github.com/nalinrajendran/eco-agri-bot

https://github.com/jayeshbhandarkar/AgriGenius

Response Assessment:
– Satisfied/Unsatisfied: Satisfied
– Additional Comments: The table includes both benchmarks and tools, but it still gives a good overview. And I meant overview in terms of features, which could be more granular, such as specifying the types of phenotyping tasks or plant trait assessments supported.

Response: Thank you for your positive assessment of our revision and for your helpful additional comment.

We agree that more granular details are valuable. However, such information is not always reported in the corresponding papers or code repositories of the included methods. In addition, to maintain readability, we have limited the number of table columns and ensured a sufficiently large font size.

Nevertheless, in this revision, we have further expanded the content of the Primary Scope and Key Ability columns to provide more granular features of each listed method, where applicable. Please refer to Supplementary Table 1.

Reviewer #4 (Remarks to the Author):

I co-reviewed this manuscript with one of the reviewers who provided the listed reports. This is part of the Nature Communications initiative to facilitate training in peer review and to provide appropriate recognition for Early Career Researchers who co-review manuscripts.

Reviewer #4 (Remarks on code availability):

I co-reviewed this manuscript with one of the reviewers who provided the listed reports. This is part of the Nature Communications initiative to facilitate training in peer review and to provide appropriate recognition for Early Career Researchers who co-review manuscripts.

Response: We sincerely thank you for your thoughtful review on both our manuscript and code repository. Our code repository is available at https://github.com/vios-s/PhenoAssistant/ (latest version: branch v2), which includes implementation of PhenoAssistant, together with the chat logs of case studies and evaluations.

Responses to Reviewers' Comments

To Editor and All Reviewers:

We sincerely thank you for your time and effort in reviewing our revised manuscript and response letter. We appreciate your constructive comments, which have helped us to improve our work. We are happy that all reviewers were satisfied with our responses.

Reviewer #3 (Remarks to the Author):

I am happy with the careful responses from the authors, no further comments.

Response: We appreciate the time and effort you have devoted to improving our manuscript. We are glad that our revisions have addressed all your concerns.
